# Differential regulation of fibroblast growth factor receptor 1 trafficking and function by extracellular galectins

**DOI:** 10.1186/s12964-019-0371-1

**Published:** 2019-06-17

**Authors:** Marika Kucińska, Natalia Porębska, Agata Lampart, Marta Latko, Agata Knapik, Małgorzata Zakrzewska, Jacek Otlewski, Łukasz Opaliński

**Affiliations:** Faculty of Biotechnology, Department of Protein Engineering, University of Wroclaw, Joliot-Curie 14a, 50-383 Wroclaw, Poland

**Keywords:** Cell proliferation, Galectins, FGFR1, Receptor clustering, Signaling, Apoptosis

## Abstract

Fibroblast growth factor receptors (FGFRs) are integral membrane proteins that transmit signals through the plasma membrane. FGFRs signaling needs to be precisely adjusted as aberrant FGFRs function is associated with development of human cancers or severe metabolic diseases. The subcellular localization, trafficking and function of FGFRs rely on the formation of multiprotein complexes. In this study we revealed galectins, lectin family members implicated in cancer development and progression, as novel FGFR1 binding proteins. We demonstrated that galectin-1 and galectin-3 directly bind to the sugar chains of the glycosylated extracellular part of FGFR1. Although both galectins compete for the same binding sites on FGFR1, these proteins elicit different impact on FGFR1 function and cellular trafficking. Galectin-1 mimics fibroblast growth factor as it efficiently activates FGFR1 and receptor-downstream signaling pathways that result in cell proliferation and apoptotic evasion. In contrast, galectin-3 induces extensive clustering of FGFR1 on the cell surface that inhibits constitutive internalization of FGFR1. Our data point on the interplay between extracellular galectins and FGFRs in the regulation of cell fate.

**Electronic supplementary material:**

The online version of this article (10.1186/s12964-019-0371-1) contains supplementary material, which is available to authorized users.

## Background

Fibroblast growth factor receptors (FGFRs) are receptor tyrosine kinases (RTKs) that together with extracellular fibroblast growth factors (FGFs) transmit signals through the plasma membrane. The FGFRs-FGFs signaling is important for angiogenesis, regulation of metabolism and tissue repair [1–3]. The dysregulation of FGFRs leads to developmental disorders and cancer [4–7]. FGFR1 is overexpressed in lung cancers, breast cancers, osteosarcomas and in solid tumors like head and neck cancers, and it is associated with poor patient prognosis [8–16]. FGFR1 is composed of an N-terminal extracellular region built of three Ig domains: D1, D2 and D3, from which D2 and D3 are involved in FGFs binding, a single transmembrane span and an intracellular protein kinase domain. Binding of FGFs to the extracellular part of FGFR1 stimulates receptor dimerization and induces conformational changes resulting in the activation of FGFR1 and propagation of signals [2, 17, 18]. The extracellular part of FGFRs is N-glycosylated at several positions and these modifications may influence FGFRs interaction with FGFs [19, 20].

Galectins comprise a family of 15 carbohydrate-binding proteins that via interaction with galactose-containing glycoconjugates regulate pivotal cellular processes like signaling, apoptosis, differentiation, immunity, migration and gene transcription [21–23]. The pattern and level of galectins expression varies between cell types and tissues [22, 24]. Galectins are ubiquitous in the extracellular environment, which they reach via non-classical secretion [25–27]. At the cell surface galectins regulate spatial distribution, trafficking and function of glycoproteins, including three RTK members: EGFR, VEGFR and IR [28–36].

Here, we identified extracellular galectin-1 and galectin-3 as interaction partners of FGFR1 by affinity purification and mass spectrometry. We provide evidence for the novel mode of FGFR1 regulation by extracellular oligomeric lectins, which by binding to the sugar chains on FGFR1 differentially adjust spatial distribution and function of this receptor. Since both FGFRs and galectins are strongly implicated in cancer, our results may be of high importance for designing of effective anticancer therapies.

## Methods

### Antibodies and reagents

The primary antibodies directed against FGFR1 (#9740), ERK1/2 (#9102), phospho-tyrosine (pTyr; #9411), Lamin-A (LamA; #4777) and phospho-ERK1/2 (pERK1/2; #9101) were from Cell Signaling. The primary antibodies against phospho-FGFR (pFGFR; #06–1433) were from Merck Millipore. The primary antibodies: anti-FGFR1 (sc-121), anti-Hsp60 (sc-136,291), anti-α-Adaptin 1/2 (sc-10,761) were from Santa Cruz Biotechnology. Anti-tubulin primary antibody (#T6557) and anti-galectin-3 antibody (#HPA003162) were from Sigma-Aldrich. Anti- human IgG (Fc) antibody coupled to HRP (#4–10-20) and goat anti-rabbit AlexaFluor-488 conjugated antibody (#ab150077) were from Abcam. The primary antibodies against VAPA (#STJ26070), Rsk-1 (STJ95547), galectin-1 (STJ93201) were from St John’s Laboratory. Anti-His-Tag primary antibodies (#MAB050) were from R&D Systems. The primary antibodies against EDIL-3 were from Abcam. The rabbit polyclonal antibodies against FGF1 were generated by Davids Biotechnologie GmbH by immunization of rabbits with purified FGF1. Secondary antibodies were from Jackson Immuno-Research Laboratories.

Protein A Sepharose, Glutathione Sepharose and Heparin Sepharose resins were from GE Healthcare. Ni-NTA agarose was from QIAGEN and Streptavidin Agarose affinity resin was from Thermo Fisher Scientific. Geneticin (G-418) and Nonidet P-40 were from BioShop. PD173074, MESNA, heparin, PNGase F, digitonin and Pitstop2 were from Sigma-Aldrich. Protease Inhibitors Cocktail was from Roche. Streptavidin-AlexaFluor-550 (SA-550), NucBlue Live, EZ-Link™ Sulfo-NHS-SS-Biotin, DyLight488 and DyLight550 were from Thermo Fisher Scientific.

### Cells

U2OS cells (human osteosarcoma, ATCC #HTB-96), U2OSR1, U2OS-SBP-R1 (U2OS cells stably transfected with SBP-FGFR1) and U2OS-R1-K514R cells were a kind gift of Dr. E.M. Haugsten from the Institute for Cancer Research, Oslo University Hospital. Cells were cultivated in Dulbecco’s Modified Eagle’s Medium (DMEM, Biowest) supplemented with 10% fetal bovine serum (Thermo Fisher Scientific) and antibiotics (100 U/ml penicillin, 100 μg/ml streptomycin). For U2OS-R1, U2OS-R1-K514R and USOS-SBP-R1 cells growth media were additionally supplemented with geneticin (0.2–1 mg/ml). NIH3T3 (murine embryonic fibroblasts, ATCC #CRL-1658) were grown in DMEM supplemented with 2% bovine serum (Thermo Fisher Scientific) and antibiotics (100 U/ml penicillin, 100 μg/ml streptomycin). All cells were cultivated in 5% CO_2_ atmosphere at 37 °C and seeded into tissue culture plates 1 day prior start of the experiments.

### Recombinant proteins

Recombinant FGF1 and FGF2 were produced in *E. coli,* as described previously [37]. The Fc fragment of IgG and the full-length extracellular portion of FGFR1 (IIIc), FGFR2 (IIIc), FGFR3 (IIIc) and FGFR4 (FGFR1ecd-Fc, FGFR2ecd-Fc, FGFR3ecd-Fc, FGFR4ecd-Fc) were expressed in CHO cells and purified using Protein A Sepharose [38].

The expression vector allowing for production of the extracellular part of FGFR1 fused to GST (GST-FGFR1ecd) in *E. coli* was prepared using Gateway Cloning (Thermo Fisher Scientific) by recombination to pDEST15 plasmid. GST-FGFR1ecd protein was expressed in *E. coli* BL21 CodonPlus(DE3)-RIL (Agilent Technologies). Inclusion bodies containing GST-FGFR1ecd were purified by sequential washing with buffer A (50 mM Tris, 1 mM EDTA, 100 mM NaCl, 10 mM DTT, 2% Triton X-100, pH 8.0), buffer B (50 mM Tris, 1 mM EDTA, 100 mM NaCl, 10 mM DTT, pH 8.0) and buffer C (50 mM Tris, 100 mM NaCl, pH 8.0). Purified inclusion bodies were then re-suspended in 6 M guanidine hydrochloride and refolded by dilution to ice-cold PBS followed by overnight stirring. Soluble fraction was collected and GST-FGFR1ecd was recovered using Glutathione Sepharose.

Plasmids: pETM-galectin1 and pETM-galectin-3 allowing for production of recombinant galectin-1 and galectin-3 as His-Tag fusions were a kind gift of Dr. Stefanie Hauck (Research Unit Protein Science, Helmholtz Institute Munich, Germany). Recombinant galectin-1 and galectin-3 were produced in *E. coli* BL21 CodonPlus(DE3)-RIL (Agilent Technologies) and purified using Ni-NTA affinity chromatography and gel filtration on HiPrep 16/60 Sephacryl S-100 column.

Fluorescently labeled proteins: FGF2-DL550, galectin-1-DL-488 and galectin-3-DL-488 were obtained by chemical labeling of recombinant proteins with DyLight protein labeling kits (Thermo Fisher Scientific). Proteins were labeled according to protocol provided by the manufacturer.

### Affinity purification of SBP-FGFR1 complexes

U2OSR1 cells (control, producing untagged FGFR1) and U2OS-SBP-R1 cells (producing SBP-FGFR1) (80 × 10^6^ cells for each cell line) were serum starved for 4 h. For experiments with growth factor stimulation, cells were pretreated with 30 μM Pitstop2 for 15 min and then treated with FGF1 (100 ng/ml) and heparin 10 U/ml for 15 min.

Cells were washed with PBS and lysed with Lysis Buffer LB (50 mM Tris, 150 mM NaCl, 1 mM EDTA, 0.1% Nonidet P-40, 1 mM PMSF, Protease Inhibitors Cocktail, pH 8.0). Lysate was briefly sonicated and subjected to clarifying spin (14,000 rpm, 10 min, 4 °C). Supernatant was incubated overnight at 4 °C with 150 μl of LB-equilibrated Streptavidin Agarose resin with end over end shaking. Beads were washed with washing buffer WB (50 mM Tris, 150 mM NaCl, 1 mM EDTA, pH 8.0) and with PBS. Beads containing bound proteins were subsequently subjected to mass spectrometry-based protein identification. Each experiment was performed in 5 repeats.

### Pull down experiments

#### Streptavidin-agarose pull down

U2OSR1 cells (control, producing untagged FGFR1) and U2OS-SBP-R1 cells (producing SBP-FGFR1) (2 × 10^6^ for each cell line per isolation) were washed with PBS and lysed with LB. Lysate was briefly sonicated and subjected to clarifying spin (14,000 rpm, 10 min, 4 °C). Supernatant was incubated for 3 h at 4 °C with LB-equilibrated Streptavidin-Agarose resin with end over end shaking. Beads were washed with washing buffer WB and PBS. Proteins were eluted with SDS-PAGE sample buffer or with LB containing 4 mM biotin (for elution under native conditions). Proteins were separated using SDS-PAGE or BN-PAGE and visualized with CBB dye or analyzed with western blotting. For analysis of FGFR1 activation cells were subjected prior to pull down to 4 h or overnight starvation followed by stimulation with FGF1 (100 ng/ml) and heparin (10 U/ml), galectin-1 (5 μg/ml) or galectin-3 (5 μg/ml) for 15 min. For analysis of FGFR1-FGF1 interaction cells were incubated with 300 ng/ml FGF1 in the presence of 20 U/ml heparin.

#### Ni-NTA agarose pull down

Purified recombinant galectin-1 or galectin-3 (10 μg), bearing His-Tag at the N-terminus, were bound to Ni-NTA in 50 mM Tris, 150 mM NaCl, pH 7.4. U2OS-R1 cells were lysed in LB and clarified lysate was incubated with Ni-NTA agarose-bound proteins for 2 h at 4 °C with end over end shaking. Beads were extensively washed with PBS and bound proteins were eluted with SDS-PAGE sample buffer. Proteins were separated by SDS-PAGE and analyzed with western blotting.

### BLI measurements

To analyze the interaction between FGFR1-FGFR4 and galectin-1 and galectin-3 bio-layer interferometry (BLI) was applied using Octet RED K2 system (ForteBio). The extracellular part of FGFR1c, FGFR2c, FGFR3c and FGFR4 (FGFR1ecd-Fc-FGFR4ecd-Fc; 10 μg/ml) was chemically immobilized on AR2G biosensors or Protein-A sensors and subsequently interaction of FGFR1 with galectin-1 or galectin-3 (10–30 μg/ml) was analyzed. Values from empty reference sensor were subtracted. For measurements of binding constants sensor-immobilized FGFR1ecd.Fc was incubated with various concentrations of galectin-1 and -3 (from 2.8 μM to 11.4 μM). Values from the control empty sensors were subtracted and heterogeneous ligand (2:1) model was used for fitting using Data Analysis 11 Software (Fortebio).

To study the impact of FGFR1 glycosylation on receptor interaction with galectin proteins, FGFR1ecd-Fc was de-glycosylated with PNGase F. To this end, FGFR1ecd-Fc (0.5 mg/ml) was incubated with PNGase F (0.3 U/ml) for 4 h at 37 °C. The enzymatic removal of sugar chains from FGFR1ecd-Fc was monitored with SDS-PAGE. FGFR1ecd-Fc and de-glycosylated FGFR1ecd-Fc (10–30 μg/ml) were immobilized on Protein-A biosensors and incubated with galectin-1 or galectin-3 (30–60 μg/ml). Alternatively, GST-FGFR1ecd (10 μg/ml) was immobilized on AR2G biosensors and incubated with galectin proteins (10 μg/ml) or with FGF2 (10 μg/ml). For competitive binding experiments FGFR1ecd-Fc (10 μg/ml) was immobilized on Protein-A biosensors. Next, sample biosensors were incubated with galectin-1 or galectin-3 (30 μg/ml) to saturate FGFR1ecd-Fc, whereas reference biosensor was incubated in the buffer only. Next, biosensors were incubated with FGF2 (10 μg/ml), galectin-1 (10 μg/ml) or galectin-3 (10 μg/ml).

### Fluorescence microscopy

For analysis of the internalization of SBP-FGFR1, U2OS-SBP-R1 cells were incubated with Streptavidin-AlexaFluor-555 (5 μg/ml) for 1 h on ice. Next, cells were incubated with FGF1 (200 ng/ml) and heparin (20 U/ml) for 2 h at 37 °C or kept on ice (control). Cells were washed with PBS, nuclei were stained with NucBlue Live dye, fixed with 4% paraformaldehyde and analyzed with fluorescence microscopy. For quantitative analysis of SBP-FGFR1 internalization intracellular fluorescence of Streptavidin-AlexaFluor-555 was measured in 5 fields of view (at least 50 cells) in triplicate using ZEN 2.3 software.

For analysis of galectin-1-DyLight488 and galectin-3-DyLight488 co-localization with SBP-FGFR1, U2OS-SBP-R1 cells were pre-incubated with Streptavidin-AlexaFluor-555 (4 μg/ml) for 5 min at RT to label SBP-FGFR1. Next, fluorescently labeled galectin proteins were added to the cells at concentrations 5–10 μg/ml and cells were incubated for 1 h at 37 °C. Cells were washed with PBS, nuclei were stained with NucBlue Live dye, cells fixed in 4% paraformaldehyde and analyzed with fluorescence microscopy. For analysis of FGF2-DyLight555 internalization, U2OS-R1 cells were preincubated for 30 min with non-labeled galectins and incubated for 30 min with FGF2-DyLight555 (2 μg/ml) in the presence of 20 U/ml heparin.

Wide-field fluorescence microscopy was carried out using a Zeiss Axio Observer Z1 fluorescence microscope (Zeiss). Images were taken using LD-Plan-Neofluar 40×/0.6 Korr M27 objective and Axiocam 503 camera. Streptavidin-AlexaFluor-555 and FGF2-DyLight555 signal was visualized with a 540/552 nm bandpass excitation filter and a 575/640 nm bandpass emission filter. Galectin-1-DyLight488 and galectin-3 DyLight488 signal was visualized with a 450/490 nm bandpass excitation filter and a 550/590 nm bandpass emission filter. NucBlue Live signal was visualized with a 335/383-nm bandpass excitation filter and a 420/470-nm emission filter. Image analysis was carried out using ZEN 2.3 and ImageJ.

### FGFR1 activation and downstream signaling cascades

To study the impact of extracellular galectin-1 and galectin-3 on the activation of FGFR1, serum starved cells were stimulated for 15 min with FGF1 (50 ng/ml) in the presence of heparin (10 U/ml) or with various concentrations of recombinant galectin-1 (0.5–10 μg/ml) or galectin-3 (0.5–10 μg/ml). Cells were lysed in SDS-PAGE sample buffer and subjected to SDS-PAGE and western blotting. In selected experiments cells were pre-incubated for 15 min with 100 nM PD173074 that was kept throughout the experiment.

### Cell apoptosis and proliferation

#### Annexin assay

NIH3T3 cells were subjected to serum starvation (DMEM without serum) for 24 h to induce apoptosis and then treated with FGF1 (200 ng/ml) or galectins-1 and galectin-3 (10 μg/ml) and heparin (10 U/ml) in the presence or absence of FGFR inhibitor (100 nM PD173074) for 24 h. Next, the cells were harvested and stained with annexin V and 7AAD using Muse Annexin V and Dead Cell Assay Kit (Merck) and analyzed as described before [39]. Experiments were performed three times (*n* = 3) with at least three replicates in each experiment.

#### Caspase-3/7 activity assay

NIH3T3, U2OS-R1 or U2OS-R1-K514R cells were subjected to serum starvation for 24 h to induce apoptosis. Then cells were treated with FGF1 (200 ng/ml) or galectin-1 and -3 (10 μg/ml) and heparin (10 U/ml) in the presence or absence of FGFR inhibitor (100 nM PD173074) for 16 h. The FGFR inhibitor was added 15 min prior to stimulation and were kept throughout the experiment. Next, caspase-3/7 activity was determined using Caspase-Glo 3/7 Assay (Promega) according to the manufacturer’s protocol. Caspase 3/7 activity was then normalized to cells untreated with FGF1 and galectins, and denoted as relative caspase-3/7 activity. All experiments were performed three times (*n* = 3) with at least three replicates in each experiment.

#### Cell proliferation

NIH3T3 were cultured in serum-free medium (DMEM) for 48 h. Cells were subsequently treated with galectin-1, galectin-3 (1–20 μg/ml) or FGF1 (1 ng/ml) with 10 U/ml heparin in the presence or absence of 100 nM PD173074. Cells were incubated at 37 °C, 5% CO_2_ for 48 h and cell proliferation was determined with Alamar Blue (Thermo Fisher Scientific).

### Mass spectrometry experiments

Detailed information about mass spectrometry experiments can be found in Additional file 1.

### Blue native PAGE (BN-PAGE)

For Blue Native PAGE (BN-PAGE) purified extracellular region of FGFR1 (FGFR1ecd-Fc (2 μg) was incubated with recombinant galectin-1 or galectin-3 (2–5 μg) in PBS for 15 min at RT and proteins were separated on 4–13% gradient BN-PAGE gels [40]. Proteins were transferred onto PVDF membrane and detected with anti-Fc antibodies, stripped and detected with anti-galectin-3 and anti-galectin-1 antibodies.

### Internalization of cell surface proteins

Analysis of the internalization of cell surface proteins was performed according to [41]. Serum starved U2OSR1 cells were cooled down on ice, washed with PBS and cell surface proteins were reversibly biotinylated with EZ-Link™ Sulfo-NHS-SS-Biotin (1 mg/ml) for 30 min on ice. Cells were washed with ice cold PBS and excessive EZ-Link™ Sulfo-NHS-SS-Biotin was quenched with 50 mM Tris, pH 8.0. For quantitative analysis of cell surface proteins internalization, cells after reversible biotinylation were left untreated (control), or incubated for 2 h at 37 °C with FGF1 (200 ng/ml, heparin 20 U/ml). Next, cells were washed extensively with PBS, fixed in 2% paraformaldehyde for 40 min, permeabilized with 0.1% Tween 20 in PBS for 30 min, blocked with 3% BSA in PBS and internalized cell surface proteins were labeled with Streptavidin-AlexaFluor-555 (2 μg/ml) in PBS supplemented with 3% BSA for 30 min. Cells were washed with PBS and analyzed with fluorescence microscopy. Intracellular fluorescent signal was measured in cells from at least 5 fields of view (at least 20 cells) using ZEN 2.3 software.

To demonstrate that intracellular signal represents internalized biotinylated cell surface proteins, selective removal of non-internalized biotin residues was performed with membrane-impermeable reductant – MESNA. To this end, biotinylated cells were were cooled down on ice to stop endocytosis and biotin was removed from non-internalized cell surface proteins by washing the cells in dark with 300 mM MESNA in 50 mM Tris, 100 mM NaCl, 1 mM EDTA, 0.2% BSA, pH 8.6. Cells were subsequently washed with PBS, then incubated with PBS containing iodoacetamide (5 mg/ml) for 10 min on ice.

## Results

### FGFR1 interactome reveals galectin family members as novel FGFR1 binding partners

The cellular trafficking and function of FGFR1 depends on the receptor interaction with specific partner proteins. Up to date a few attempts to identify proteins that bind FGFR1 were reported [42, 43]. Here, we sought to identify novel proteins that adjust FGFR1 function and cellular distribution.

**Fig. 1:**
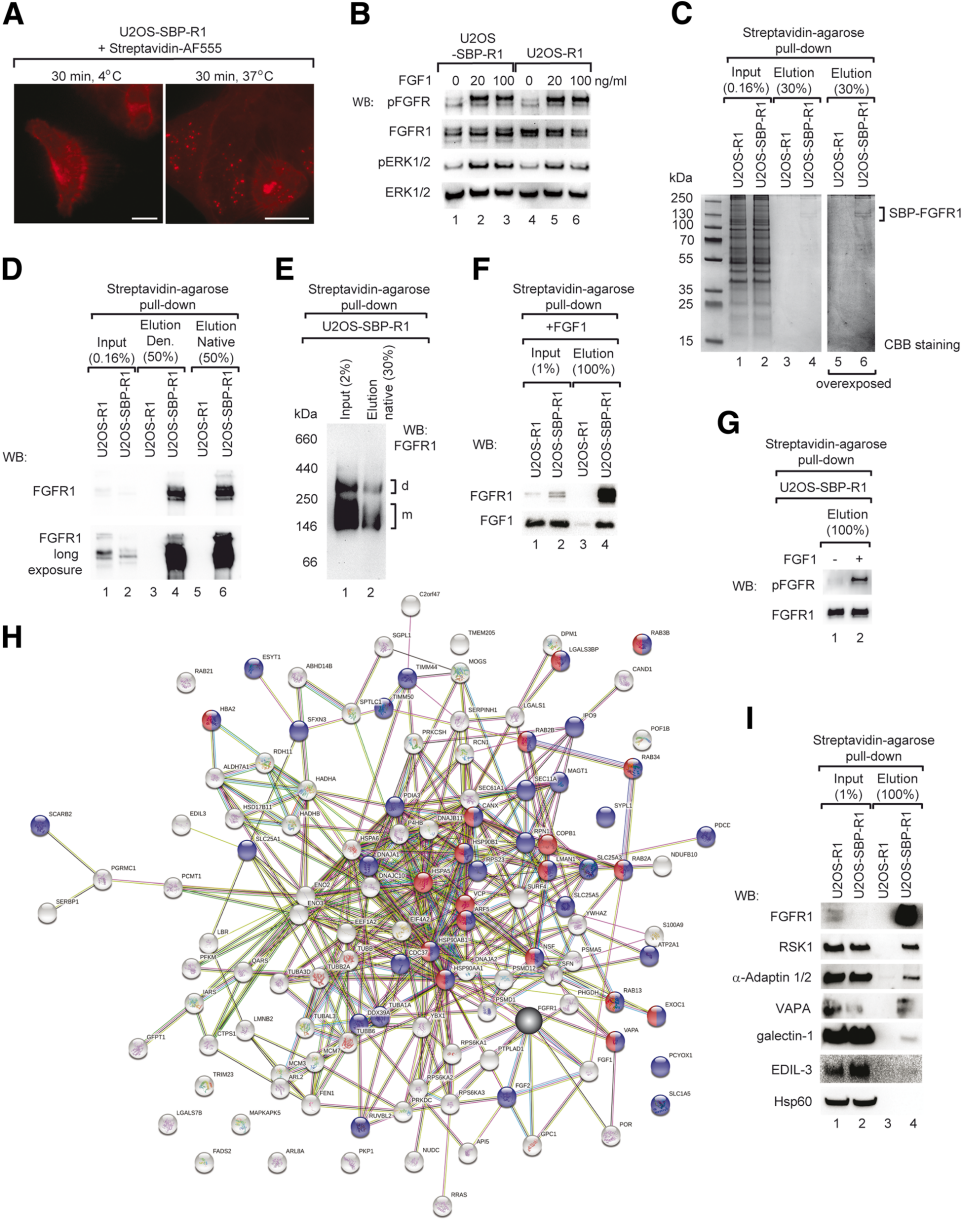
Identification of FGFR1 partner proteins. **a** Cellular localization of SBP-FGFR1. U2OS-SBP-R1 cells were incubated with Streptavidin-AlexaFluor-555 and kept either at 4 °C or 37 °C. Cells were fixed and analyzed with fluorescence microscopy. Scale bars represent 50 μm. **b** Serum starved U2OS-R1 and U2OS-SBP-R1 cells were stimulated for 15 min with FGF1 (20–100 ng/ml) and heparin (10 U/ml). Cells were lysed and activation of cellular signaling was assessed with western blotting. **c** Purification of full-length SBP-FGFR1 from model mammalian cells. U2OS-R1 and U2OS-SBP-R1 cells were lysed under mild conditions and SBP-FGFR1 was recovered by affinity purification using streptavidin-agarose resin. Proteins were eluted with sample buffer and separated with SDS-PAGE and stained with CBB. **d** Western blotting analysis of SBP-FGFR1 affinity purification from U2OS-SBP-R1 cells. Denaturing elution was performed with SDS-PAGE sample buffer, while elution under native conditions was carried out with 4 mM biotin. **e** BN-PAGE analysis of SBP-FGFR1 purification. SBP-FGFR1 isolated from U2OS-SBP-R1 cells under non-denaturing conditions was subjected to 4–13% BN-PAGE. The migration of SBP-FGFR1 was analyzed with western blotting. **f** SBP-FGFR1 interacts with FGF1. U2OS-SBP-R1 and U2OS-R1 cells were incubated with FGF1, cells were washed, bound proteins were eluted with SDS-PAGE sample buffer and analyzed with western blotting. **g** Streptavidin-agarose pull down allows for purification of activated SBP-FGFR1. Serum starved U2OS-SBP-R1 cells were treated for 15 min with FGF1 (200 ng/ml) and heparin (20 U/ml). Next, cells were lysed, SBP-FGFR1 was isolated via affinity purification and activation of the receptor was assessed with western blotting. **h**. SBP-FGFR1 interaction network identified in affinity purification followed by mass spectrometry. Proteins were classified using STRING (http://www.string-db.org/). Blue spheres represent proteins involved in cellular protein transport, while red spheres depict proteins implicated in vesicle trafficking. **i** Verification of MS experiments. U2OS-SBP-R1 and U2OS-R1 cells were lysed and co-purification of selected proteins with SBP-FGFR1 was determined with western blotting

We used model U2OS-SBP-R1 cells producing streptavidin-binding peptide (SBP)-tagged FGFR1 (SBP-FGFR1) and U2OS-R1 cells stably expressing non-tagged FGFR1 as a control [43]. At steady state conditions FGFR1 is localized to the plasma membrane [44–46]. Accordingly, at conditions were receptor endocytosis is blocked (4 °C) SBP-FGFR1 was localized to the cell surface as demonstrated with fluorescence microscopy (Fig. 1a). When SBP-FGFR1 was pre-labeled with streptavidin-AF555 at 4 °C and then cells were incubated at 37 °C to allow for internalization, punctate structures representing internalized SBP-FGFR1 were observed (Fig. 1a). Growth factor binding leads to FGFR1 trans-activation and effectively stimulates internalization of FGF-FGFR1 complexes [17, 46, 47]. To study whether FGF1 binding to SBP-FGFR1 accelerates uptake of the receptor, U2OS-SBP-R1 cells were pre-labeled with streptavidin-AF555 and then incubated in the presence or absence of FGF1. The efficiency of FGFR1 internalization was determined by fluorescence microscopy. FGF1 increased internalization of SBP-FGFR1 by about 40% (Additional file 1: Figure S1). Interestingly, at the same conditions the efficiency of total cell surface proteins internalization was independent of FGF1 (Additional file 1: Figure S1). These data indicate that binding of FGF1 to SBP-FGFR1 specifically stimulates internalization of FGFR1. In agreement with these findings, the stimulation of U2OS-SBP-R1 cells with FGF1 led to the activation of the FGFR1 and receptor-downstream kinase ERK1/2, similarly to the control U2OS-R1 cells, as demonstrated by western blotting (Fig. 1b). These data show that SBP-tagged FGFR1 retained biological activity and subcellular trafficking.

Next, we established a protocol for efficient isolation of full length SBP-FGFR1 from the plasma membrane of U2OS-SBP-R1 cells under native conditions using affinity chromatography with streptavidin-agarose affinity matrix. The elution fractions from U2OS-SBP-R1 isolation contained proteins of over 100 kDa that were absent in the control purification from U2OS-R1 cells (Fig. 1c, lanes 3–6). The amounts of purified full length SBP-FGFR1 were sufficient for protein visualization with Coomassie Brilliant Blue (CBB) dye (Fig. 1c, lanes 4 and 6). We confirmed the identity of isolated SBP-FGFR1 with western blotting using FGFR1-specific antibodies (Fig. 1d). The SBP tag allowed for elution of purified SBP-FGFR1 and its complexes under mild, non-denaturing conditions using biotin (Fig. 1d, lanes 5 and 6). We and others have recently demonstrated that in the absence of FGF1 FGFR1 is present in high molecular weight complexes that likely represent unliganded receptor dimers [47–51]. To study whether isolated SBP-FGFR1 retained its ability to form high molecular weight complexes we have performed streptavidin-agarose pull down and eluted bound proteins with biotin. Protein complexes were separated with blue native protein acrylamide gel electrophoresis (BN-PAGE) and detected with western blotting. SBP-FGFR1 migrates as two major bands on BN-PAGE: broad band of about 150 kDa, which likely represents different glycoforms of receptor monomers and high molecular weight band of molecular weight over 300 kDa probably reflecting receptor dimers and/or complexes with partner proteins (Fig. 1e, lane 1). All these complexes are present in the elution fractions from streptavidin-agarose pull down from U2OS-SBP-R1 cells, demonstrating that isolated SBP-FGFR1 retained its native structure and ability to interact with itself and/or with partner proteins (Fig. 1e, lane 2).

To validate that established system is suitable for identification of proteins interacting with FGFR1, we incubated U2OS-R1 cells and U2OS-SBP-R1 cells with FGFR1 ligand, FGF1. Cells were extensively washed to remove non-specific interactions, lysed and subjected to streptavidin agarose pull down. Isolation of SBP-FGFR1 resulted in co-purification of FGF1 (Fig. 1f). Moreover, treatment of serum-starved U2OS-SBP-R1 cells with FGF1 followed by streptavidin-agarose pull down allowed for purification of activated SBP-FGFR1, as demonstrated with antibodies recognizing phosphorylated Tyr653 and Tyr654 of FGFR1 (pFGFR) (Fig. 1g). These data demonstrate that our protocol allows for isolation of full length FGFR1 from the plasma membrane of model cells and constitutes a suitable experimental model for identification of FGFR1 partner proteins.

Next, we sought to identify proteins that interact with FGFR1 at conditions where internalization of FGFR1 is induced by FGF1. To this end, U2OS-R1 and U2OS-SBP-R1 cells were serum starved, pretreated with Pitstop-2 (inhibitor of clathrin-mediated endocytosis) to decelerate receptor endocytosis and incubated with FGF1 for 15 min to initiate FGFR1 internalization. Next, streptavidin agarose pull down was applied for isolation of SBP-FGFR1 and its partner proteins. Proteins specifically co-purifying with FGFR1 were identified by mass spectrometry (MS). These analyses led to identification of 123 potential FGFR1 interaction partners, from which 102 proteins were uniquely found in elution fractions from U2OS-SBP-R1 cells and 21 proteins were enriched in U2OS-SBP-R1 elutions in relation to control U2OS-R1 (Additional file 1: Table S1). Within 123 identified proteins 16 have been already known as interaction partners of FGFR1, which validates our approach (Additional file 1: Table S1). Based on GO classification majority of identified proteins were involved in metabolism (48 proteins), signal transduction (46 proteins) and organization of cell architecture (49 proteins). Interestingly, 30 of identified proteins were implicated in cellular protein transport, including proteins from Rab, Sec and galectin families (Fig. 1h).

MS data were validated with western blotting using antibodies specific to selected proteins. For verification of mass-spectroscopy-based data we selected RSK1, a known FGFR1-interaction partner [52]; vesicle-associated membrane protein-associated protein A (VAPA), a protein implicated in membrane trafficking; EGF-like repeat and discoidin I-like domain-containing protein 3 (EDIL-3), a protein interacting with integrin α_V_β_3_, a protein implicated in FGFR signaling [53–55] and galectin-1, lectin that binds glycosylated cell surface proteins [22]. As demonstrated by western blotting all of analyzed proteins were specifically co-purified with SBP-FGFR1, whereas control protein, Hsp60, absent in MS data, was not co-eluted with SBP-FGFR1 (Fig. 1i). We confirmed that galectin-1 interacts with FGFR1 by co-immunoprecipitation using NIH3T3 cells expressing endogenous levels of studied proteins (Additional file 1: Figure S2).

### Galectin-1 and 3 directly bind to the glycosylated extracellular region of FGFR1

Besides galectin-1, among putative FGFR1 interactors we also found another dimeric galectin-7 and galectin-3 binding protein. Thus, these data implicated that not only prototype dimeric galectins (galectin-1 and galectin-7), but also chimeric pentameric galectin-3 may form complexes with FGFR1. Therefore, we decided to study in detail an interplay between selected galectins and FGFR1. We detected interaction between endogenous galectin-3 and FGFR1 in NIH3T3 cells using co-immunoprecipitation (Additional file 1: Figure S2b).

**Fig. 2:**
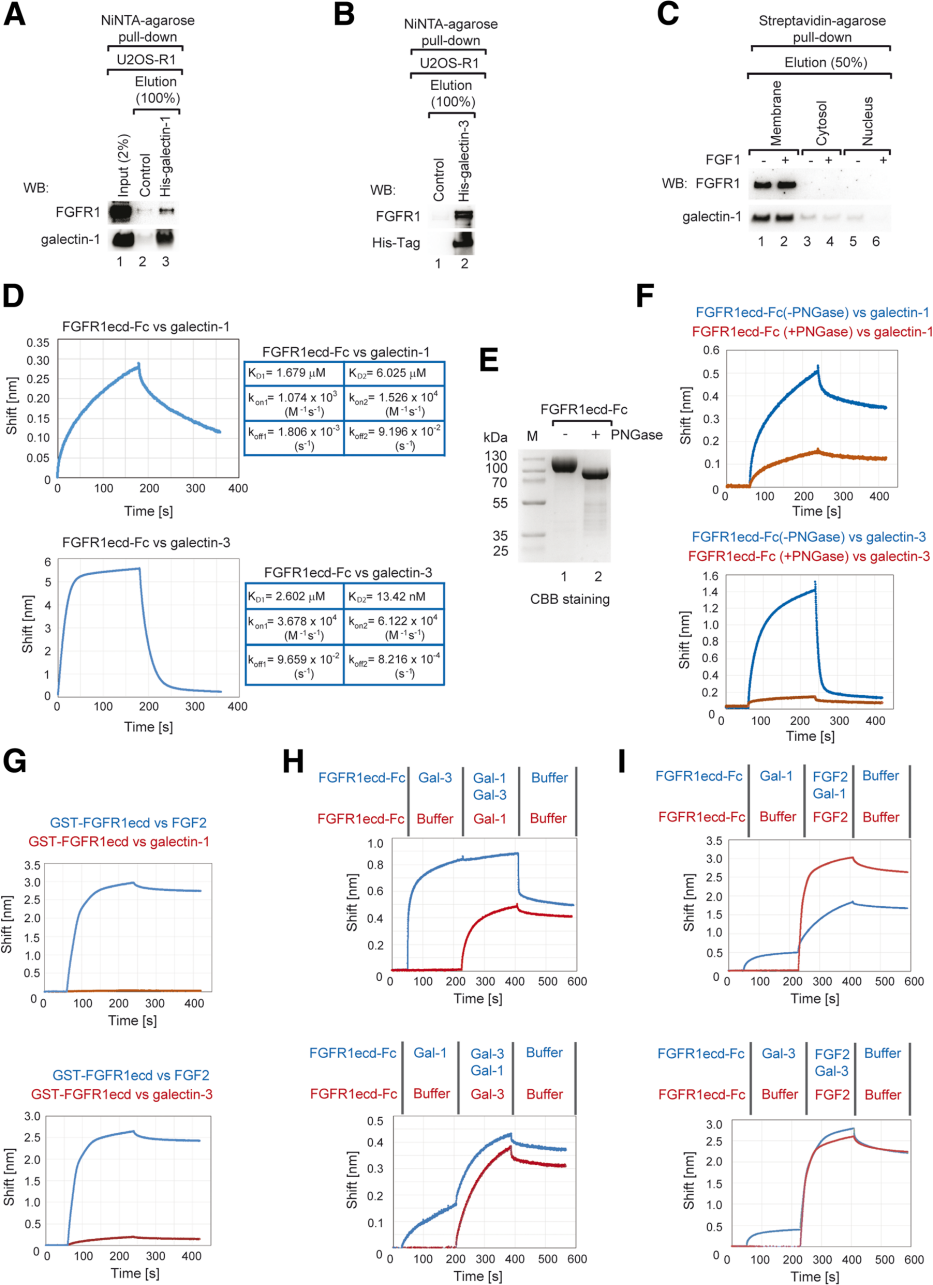
Galectin-1 and -3 directly interact with sugar chains of FGFR1 extracellular domain. **a**, **b** Purified recombinant galectin-1 and galectin-3 were bound to NiNTA and incubated with cell lysates prepared from U2OS-R1 cells. Bound proteins were analyzed with western blotting. **c** U2OS-SBP-R1 cells were treated with FGF1 (50 ng/ml), heparin (10 U/ml) and subjected to fractionation into membrane, cytosolic and nuclear fractions. Subsequently co-purification of endogenously expressed galectin-1 with SBP-FGFR1 was determined with western blotting. **d** BLI analyses of FGFR1 interactions with galectins. The extracellular part of FGFR1c (FGFR1ecd-Fc) (10 μg/ml) was immobilized of Protein-A sensors and interaction with galectin-1 and galectin-3 (10–30 μg/ml) was monitored. The binding constants for galectin-1 and galecti-3 interaction with FGFR1ecd-Fc were determined with BLI. **e** Enzymatic de-glycosylation of FGFR1ecd-Fc with PNGase. FGFR1ecd-Fc (0.5 mg/ml) was incubated with PNGase F (0.3 U/ml) for 4 h at 37 °C. The enzymatic removal of sugar chains from FGFR1ecd-Fc was monitored with SDS-PAGE. **f** BLI analysis of galectin-1 and -3 interaction with PNGase treated FGFR1ecd-Fc. FGFR1ecd-Fc and PNGase treated FGFR1ecd-Fc (10–30 μg/ml) were immobilized on sample and reference Protein A sensors, respectively, and incubated with galectin-1 and galectin-3 (10–30 μg/ml). **g** BLI analysis of galectin-1 and galectin-3 interaction with the extracellular region of FGFR1 of bacterial origin (GST-FGFR1ecd). GST-FGFR1ecd (10 μg/ml) was chemically immobilized on AR2G sensors (both sample and reference) and incubated either with FGF2 (10 μg/ml) or galectin-1 and galectin-3 (10 μg/ml). **h** Competitive binding of galectin-1 and galectin-3 to FGFR1ecd-Fc. FGFR1ecd-Fc (10 μg/ml) was immobilized on Protein-A biosensors. Sample biosensors were incubated with galectin-3 (30 μg/ml) to saturate FGFR1ecd-Fc, whereas reference biosensor was incubated in the buffer only. Next, biosensors were incubated with galectin-1 (10 μg/ml) or galectin-1 (10 μg/ml)/galectin-3 (30 μg/ml) mixture (for sensors treated first with galectin-3 to avoid galectin-3 dissociation). FGFR1ecd-Fc (10 μg/ml) was immobilized on Protein-A biosensors. Sample biosensors were incubated with galectin-1 (30 μg/ml) to saturate FGFR1ecd-Fc, whereas reference biosensor was incubated in the buffer only. Next, biosensors were incubated with galectin-3 (10 μg/ml) or galectin-3 (10 μg/ml)/galectin-1 (30 μg/ml) mixture (for sensors treated first with galectin-1 to avoid galectin-1 dissociation). **i** Influence of galectins binding on the FGFR1 interaction with FGF2. FGFR1ecd-Fc (10 μg/ml) was immobilized on Protein-A biosensors. Next, sample biosensors were incubated with galectin-1 or galectin-3 (30 μg/ml) to saturate FGFR1ecd-Fc, whereas reference biosensor was incubated in the buffer only. Next, biosensors were incubated with FGF2 (10 μg/ml)

To further confirm FGFR1 interaction with galectin-1 and galectin-3, we produced recombinant galectin-1 and -3 (Additional file 1: Figure S3a) and performed pull down experiments with resin-immobilized galectins and U2OS-R1 cell lysate. Specific co-purification of FGFR1 with galectin-1 and -3 was observed (Fig. 2a and b). To analyze whether endogenously expressed galectin-1 interacts with membrane-embedded pool of FGFR1 we performed fractionation of U2OS-SBP-R1 cells (Additional file 1: Figure S3b). Next, co-purification of galectin-1 with SBP-FGFR1 in cellular fractions (membrane, cytosol and nuclear) was assessed. SBP-FGFR1-galectin-1 complex was detected mainly in the membrane fraction, containing plasma membrane/endosomes (Fig. 2c, lanes 1 and 2). These data suggest that galectin-1 may interact with plasma-membrane localized FGFR1, likely via binding to glycosylated, extracellular part of the receptor. In agreement with above results, we have demonstrated with immunofluorescence microscopy that galectin-1 is localized to the cytosol and on the cell surface of U2OSR1 cells (Additional file 1: Figure S3c). Interestingly, efficiency of galectin-1 co-purification by SBP-FGFR1 was independent of FGF1 (Fig. 2c).

The extracellular portion of FGFR1 is N-glycosylated at several positions [19, 20]. We produced a full-length extracellular part of FGFR1 (isoform IIIc) as a fusion with the Fc region of human IgG (FGFR1ecd-Fc) in the CHO cells, which allow for receptor glycosylation [38]. Biolayer interferometry (BLI) with biosensor-immobilized FGFR1ecd-Fc and recombinant galectin-1 or galectin-3 revealed a direct interaction between these proteins (Fig. 2d). The measurements of galectin-1 and galectin-3 affinities for FGFR1ecd-Fc revealed that these proteins display two types of interactions with the receptor. Galectin-3, in comparison to galectin-1 displays higher affinity towards FGFR1ecd-Fc, which results from higher association rates than the ones observed for galectin-1 (Fig. 2d) In addition, we found that both galectins bind to the extracellular regions of other three FGFR members (FGFR2-FGFR4) (Additional file 1: Figure S3d and e).

To study whether galectin-1 and -3 bind to the sugar chains present on the extracellular region of FGFR1 we utilized two different strategies. First, we enzymatically de-glycosylated FGFR1ecd-Fc (Fig. 2e) and analyzed its interaction with galectins using BLI. Enzymatic removal of sugar chains from FGFR1ecd-Fc strongly decreased its interaction with galectin-1 and -3 (Fig. 2f). In the second approach, we produced extracellular part of FGFR1c in *E. coli* as Glutathione-S-Transferase fusion (GST-FGFR1ecd). As GST-FGFR1ecd is of bacterial origin, this protein is fully devoid of eukaryotic post-translational modifications. BLI analyses revealed that GST-FGFR1ecd was unable to interact with galectin-1 and -3, while retaining its proper conformation, which was verified by FGF2 binding (Fig. 2g). We used FGF2 in BLI experiments instead of FGF1, as it displays higher stability and low non-specific binding to BLI sensors as compared to FGF1.

As both galectin-1 and galectin-3 bind to the glycosylated extracellular region of FGFR1, we decided to check whether these proteins compete for the same binding sites on the receptor. Thus, we immobilized FGFR1ecd-Fc on BLI sensors and saturated it with galectin-3. Formation of FGFR1ecd-Fc-galectin-3 complex fully abolished interaction of galectin-1 with the receptor (Fig. 2h). Interestingly, saturating concentrations of galectin-1 were not able to outcompete binding of galectin-3 (Fig. 2h). The observed results are in agreement with higher association rates of galectin-3 in relation to galectin-1.

Next, we analyzed whether formation of FGFR1 complexes with either galectin-1 or galectin-3 influences receptor interaction with its ligand. To this end, extracellular region of FGFR1 (FGFR1ecd-Fc) was immobilized on BLI sensors. Sample sensors were saturated with galectin-1 or galectin-3, whereas reference sensors remained untreated. Then, sensors were incubated with fibroblast growth factor 2 (FGF2) and interaction of FGF2 with FGFR1ecd-Fc was analyzed. Binding of galectin-1 to FGFR1ecd-Fc altered FGF2 association and dissociation profiles (Fig. 2i). Interestingly, binding of galectin-3 to FGFR1ecd-Fc had no influence on receptor ability to bind FGF2 (Fig. 2i). These data demonstrate that galectin-1 and -3 compete for the same sugar chains on the extracellular region of FGFR1 but exert differential effect on receptor interaction with the growth factor. The various effect of galectin-1 and -3 on FGF2 binding to FGFR1ecd-Fc may result from their different steric impact on the availability of FGF-binding site on the receptor. Alternatively, this discrepancy can be explained by only partial overlap of galectin-1 and -3 binding sites on FGFR1.

### Galectins regulate spatial distribution of FGFR1

Galectin-1 and galectin-3 assemble into different oligomeric structures (dimers vs pentamers, respectively) [22]. Noteworthy, the extracellular part of FGFR1 contains several sugar chains [19, 20]. This suggested that galectin-1 and galectin-3 may differentially regulate organization of FGFR1 in the plasma membrane, e.g. by forming receptor lattices of distinct architecture and properties.

**Fig. 3:**
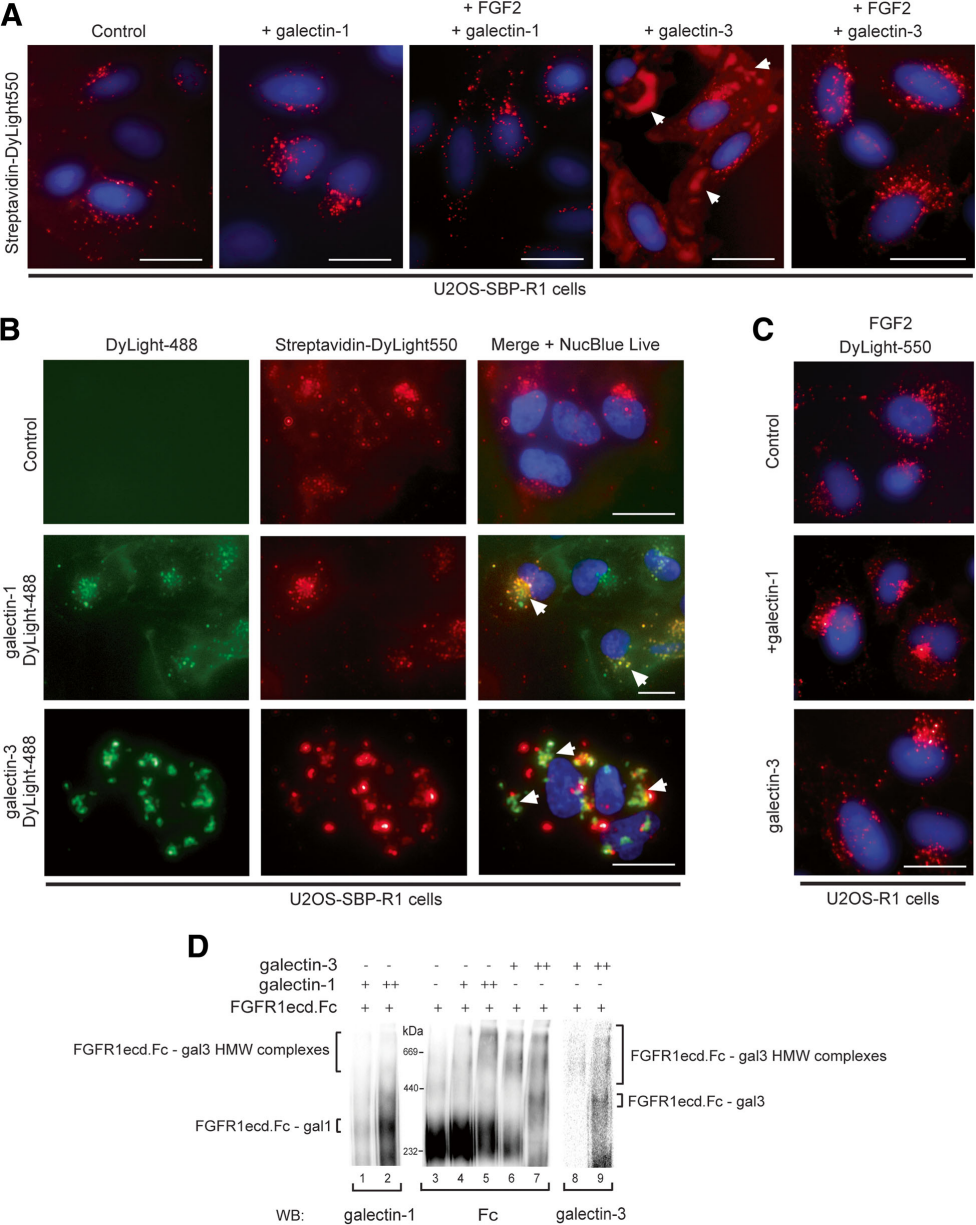
Extracellular galectins regulate spatial distribution of FGFR1. **a** Impact of galectin-1 and -3 on the cellular trafficking of SBP-FGFR1. Serum starved U2OS-SBP-R1 cells were pre-incubated with Streptavidin-AlexaFluor-555 (4 μg/ml) for 5 min on ice to label SBP-FGFR1. Next, galectins were added to the cells (10 μg/ml) and cells were in some cases treated with FGF2 (200 ng/ml) and heparin (20 U/ml) for 1 h at 37 °C. Cells were subsequently fixed, nuclei were labeled and cells were analyzed with fluorescence microscopy. Scale bars represent 50 μm. **b** Co-localization of galectins with FGFR1. For analysis of galectin-1-DyLight488 and galectin-3-DyLight488 co-localization with SBP-FGFR1, U2OS-SBP-R1 cells were pre-incubated with Streptavidin-AlexaFluor-555 (4 μg/ml) for 5 min on ice to label SBP-FGFR1. Next, fluorescently labeled galectins were added to the cells (10 μg/ml) for 1 h at 37 °C and analyzed with fluorescence microscopy. Arrows mark selected points of co-localization. **c** Influence of galectins on the trafficking of FGFR1. For analysis of the impact of galectins on FGF2 internalization, serum starved U2OS-R1 cells were incubated with galectins (10 μg/ml) for 10 min at RT and subsequently with FGF2-DyLight-550 (2 μg/ml) and heparin (20 U/ml). **d** BN-PAGE analysis of the impact of galectins on the clustering of FGFR1ecd-Fc.Purified extracellular region of FGFR1 (FGFR1ecd-Fc (2 μg)) was incubated with recombinant galectin-1 or galectin-3 (2–5 μg) in PBS for 15 min at RT and proteins were separated on 4–13% gradient BN-PAGE gels. Proteins were transferred onto PVDF membrane and detected with anti-Fc antibodies, stripped and detected with anti-galectin-3 and anti-galectin-1 antibodies

We utilized fluorescence microscopy to study the impact of galectins on FGFR1 trafficking in U2OS-SBP-R1 cells. Cells were incubated on cold with streptavidin-AF550 to label cell surface SBP-FGFR1 and then shifted for 1 h to 37 °C to allow for receptor endocytosis. SBP-FGFR1 was internalized at these conditions and was visible as numerous spots representing endosomes bearing SBP-FGFR1 (Fig. 3a). Extracellular galectin-1 caused moderate increase in the internalization of SBP-FGFR1. Similar effect was observed for the mixture of galectin-1 and FGF2 (Fig. 3a). Strikingly, extracellularly administered galectin-3 dramatically altered the cellular distribution of SBP-FGFR1. The majority of SBP-FGFR1 labeling was retained in large clusters on the cell surface while only a small fraction of receptor was internalized (Fig. 3a). We confirmed cell surface localization of SBP-FGFR1 upon galectin-3 treatment by co-localization with plasma membrane-specific dye CellMask Green (Additional file 1: Figure S4). However, SBP-FGFR1 was efficiently internalized when cells pretreated with galectin-3 were subsequently incubated with FGF2 (Fig. 3a). We fluorescently labeled galectin-1 and galectin-3 with DyLight-488 and incubated these proteins with U2OS-SBP-R1 cells, in which SBP-FGFR1 was labeled with Streptavidin-DyLight-550. The fluorescent signal of galectin-1-DyLight-488 was observed on the cell surface as well as in numerous spots that largely co-localized with SBP-FGFR1 (Fig. 3b). Galectin-3-DyLight-488 signal largely co-localized with clusters of SBP-FGFR1 (Fig. 3b). We confirmed that fluorescently labeled proteins retained the activity by analyzing their interaction with FGFR1 using BLI (Additional file 1: Figure S5). These data suggest that galectin-1-FGFR1 complexes are internalized, while galectin-3 causes extensive FGFR1 cross-linking on the cell surface, reducing FGFR1 internalization. Galectin-3-FGFR1 lattices are highly dynamic, as FGFR1 is efficiently released from the clusters by the growth factor (Fig. 3a). In agreement with these findings, internalization of FGF2 was unaffected by both galectin-1 and galectin-3 (Fig. 3c).

To study the impact of galectin-1 and galectin-3 on the oligomeric state of FGFR1 in vitro*,* we incubated recombinant galectins with FGFR1ecd-Fc and analyzed oligomeric state of the receptor with BN-PAGE. FGFR1ecd-Fc migrates as about 250 kDa protein on BN-PAGE gels which is in agreement with the calculated molecular mass of FGFR1ecd-Fc (Fig. 3d, lane 3). Binding of galectin-1 to FGFR1ecd-Fc led to the slight decrease in the mobility of FGFR1ecd-Fc, which suggests formation of FGFR1ecd-Fc – galectin-1 1:1 heterodimeric complexes (Fig. 3d, lanes 1, 2, 4 and 5). At higher concentrations of galectin-1 high molecular weight complexes composed of galectin-1 and FGFR1ecd-Fc were formed (Fig. 3d, lanes 2 and 5). Galectin-3 strongly affected oligomeric state of the FGFR1ecd-Fc, leading to formation of high molecular weight complexes (over 669 kDa) visible on top of BN-PAGE gels, containing both FGFR1ecd-Fc and galectin-3 (Fig. 3d, lanes 6–9). These data suggest that galectin-1 and galectin-3 differentially induce FGFR1 clustering.

### Differential regulation of FGFR1 activity by galectin-1 and galectin-3

**Fig. 4:**
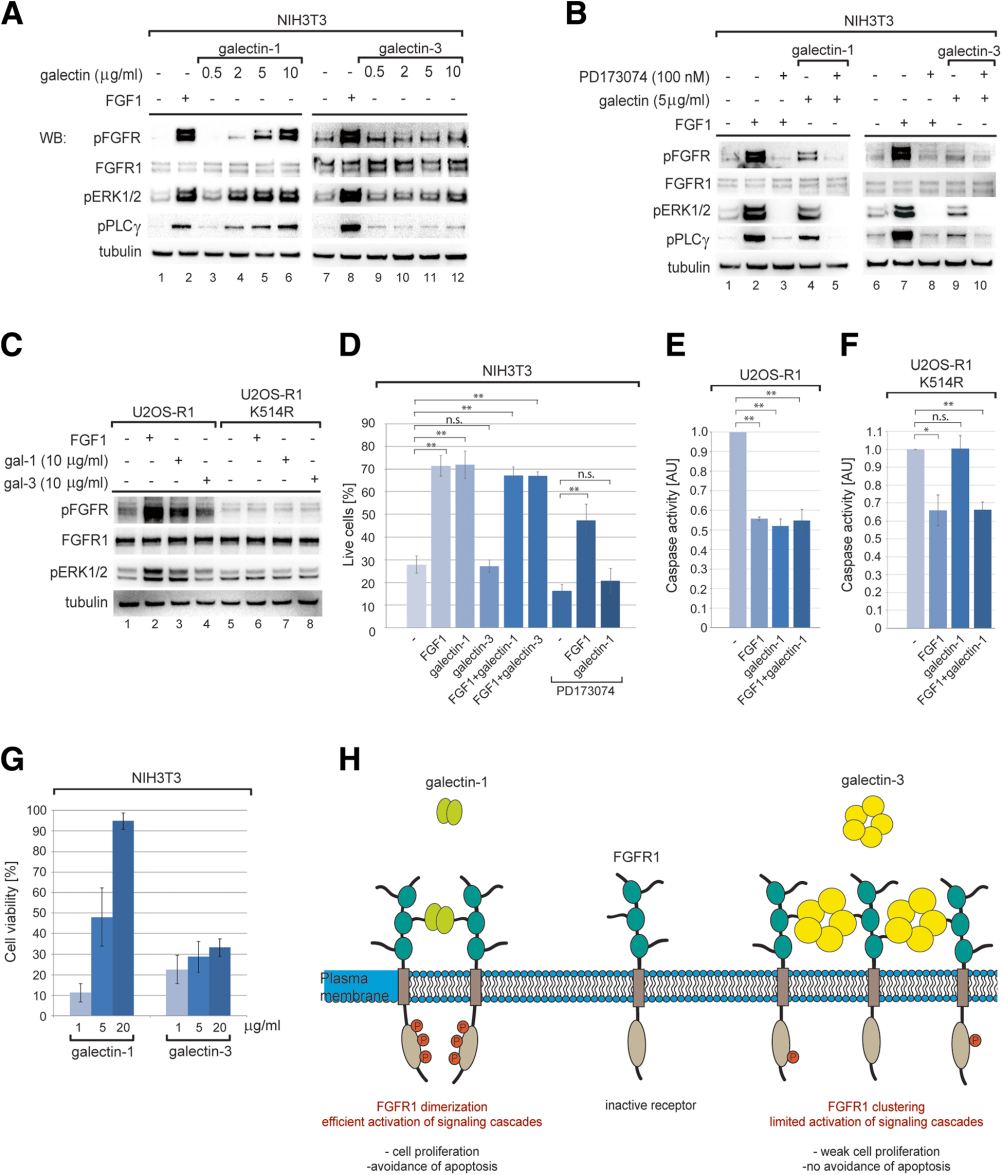
Impact of galectins on FGFR1 activity. **a** and **b** Impact of extracellular galectin-1 and -3 on the activation of FGFR1 and downstream signaling. Serum starved NIH3T3 cells were incubated for 15 min with FGF1 (50 ng/ml) and heparin (10 U/ml) or galectin-1 and -3 (0.5–10 μg/ml) either in the absence (**a**) or presence (**b**) of FGFR kinase inhibitor PD173074 (100 nM). Cells were lysed and activation of cellular signaling cascades was assessed with western blotting. **c** FGFR1 dependence of galectins signaling. Serum starved U2OS-R1 and U2OS-R1-K514R cells were incubated for 15 min with FGF1 (50 ng/ml) and heparin (10 U/ml) or galectin-1 and galectin-3 (10 μg/ml). Cellular signaling was studied with western blotting. **d** Anti-apoptotic activity of galectin-1 assessed with annexin assay. NIH3T3 cells were serum starved to induce apoptosis and treated with FGF1 (200 ng/ml) and heparin (10 U/ml), galectin-1 or galectin-3 (10 μg/ml) in the presence or absence of FGFR inhibitor (100 nM PD173074) for 24 h. Average values from three experiments +/− SD are shown. Student t-test was applied for statistical analysis (** *p* < 0.005; n.s. – not significant). **e**, **f** Anti-apoptotic activity of galectin-1 fully depends on FGFR1 kinase activity. U2OS-R1 or U2OS-R1-K514R cells were subjected to serum starvation for 24 h to induce apoptosis. Cells were treated with FGF1 (200 ng/ml) and heparin (10 U/ml) or galectin-1 (10 μg/ml) for 16 h. Next, caspase-3/7 activity was determined, normalized to cells untreated with FGF1 and galectins and denoted as relative caspase-3/7 activity. All experiments were performed three times. Average values +/− SD are shown. Student t-test was applied for statistical analysis (*p < 0.05; ** p < 0.005; n.s. – not significant). **g** Galectins induce cell proliferation. Serum starved NiH3T3 cells were treated with galectin-1 and galectin-3 (1–20 μg/ml) or FGF1 (1 ng/ml) and heparin (10 U/ml) in the presence or absence of 100 nM PD173074. Cells were incubated at 37 °C, 5% CO2 for 48 h and cell proliferation was determined with Alamar Blue. Average values from six experiments +/− SEM are shown. **h** Model of galectin-1 and galectin-3 impact on FGFR1. By binding to the sugar chains on the extracellular region of FGFR1 galectins differentially modulate membrane distribution of the receptor and its function. Dimeric galectin-1 induces formation of FGFR1 dimers/clusters that are tyrosine-phosphorylated and initiate downstream signaling resulting in cell proliferation and avoidance of apoptosis. In contrast, larger pentameric galectin-3 triggers extensive FGFR1 cross-linking on the cell surface. In these clusters FGFR1 molecules are separated from each other in orientation that does not permit receptor activation. FGFR1 lattice induced by galectin-3 retains receptor on the cell surface, downregulating constitutive internalization of the receptor in the absence of the growth factor

To study the impact of galectins on FGFR1 function we analyzed activation of FGFR1 and receptor-dependent signaling pathways using mouse NIH3T3 fibroblasts, which exhibit high levels of endogenous FGFR1 and constitute commonly used model cell line for analysis of FGFR-induced response. Galectin-1 efficiently induced phosphorylation of FGFR1 and downstream signaling proteins: kinase ERK1/2 and PLCγ in a concentration-dependent manner and to a similar extent as FGF1 (Fig. 4a, lanes 3–6). Treatment of cells with galectin-3 caused only weak phosphorylation of FGFR1 and mild activation of ERK1/2 and PLCγ (Fig. 4a, lanes 9–12). Activation of FGFR1 and ERK1/2 by galectin-1 and galectin-3 was fully blocked by PD173074, a potent and selective FGFR inhibitor (Fig. 4b, lanes 4, 5, 9 and 10). Next, we studied cellular signaling triggered by galectin-1 and -3 using U2OS-R1 cells that stably produce FGFR1 and U2OS-R1-K514R cells that express kinase-dead mutant of FGFR1. Galectin-1 efficiently activated FGFR1 and ERK1/2 in U2OS-R1 cells, while it was not able to stimulate signaling cascades in U2OS-R1-K514R (Fig. 4c, lanes 3 and 7). Similarly to NIH3T3 cells, in the case of galectin-3 only mild induction of FGFR1 and ERK1/2 was observed in U2OS-R1 cells, whereas there was no signaling in U2OS-R1-K514R cells (Fig. 4c, lanes 4 and 8). These data suggest that activation of intracellular signaling pathways (eg. ERK1/2 and PLCγ) by extracellular galectins (especially by galectin-1) occurs via FGFR1.

The FGFR1-dependent signaling protects cells from apoptosis [39]. Interestingly, anti-apoptotic activity of FGF1 and FGF2 is partially independent of FGFR1 activation and is attributed to growth factor translocation to the cytosol and nucleus [39]. Galectin-1 displayed anti-apoptotic activity to the same extent as FGF1, which is in agreement with signaling studies (Fig. 4d, Additional file 1: Figure S6a and b). Weak FGFR1-dependent signaling triggered by galectin-3 was not sufficient to protect cells from starvation-induced apoptosis (Fig. 4d, Additional file 1: Figure S6a and b). Simultaneous treatment of cells with FGF1 and galectin-1 did not alter anti-apoptotic response, which suggests that these proteins operate in the same cellular signaling pathways (Fig. 4d, Additional file 1: Figure S6a and b). In agreement with binding and trafficking studies (Fig. 2h, Fig. 3a), FGF1 was fully functional in the presence of galectin-3 (Fig. 4d, Additional file 1: Figure S6a and b). The anti-apoptotic activity of galectin-1 was fully blocked by FGFR kinase inhibitor PD173074 (Fig. 4d, Additional file 1: Figure S6a and b), while activity of FGF1 was not fully inhibited by PD173074 as FGF1 additionally protects the cells from apoptosis by translocating into the cell interior [39]. In addition, galectin-1 did not protect cells producing FGFR1-kinase dead mutant (U2OS-R1-K514R) against apoptosis (Fig. 4e and f). Our data strongly indicate that anti-apoptotic activity of galectin-1 is dependent on FGFR1 activation.

The sustained FGFR1 activation (especially ERK1/2 pathway) ultimately results in induction of cell proliferation [17]. Externally administered galectin-1 caused efficient proliferation of NIH3T3 cells in a dose-dependent manner. In contrast, galectin-3 only weakly stimulated cell division (Fig. 4g). In agreement with previous results, galectin-1-induced cell proliferation was fully abolished by PD173074 (Additional file 1: Figure S6c).

## Discussion

Overexpression of FGFRs and galectins is found in numerous tumors and is associated with poor patient prognosis [4, 24]. The indirect role of galectin-3 in the regulation of FGF2-mediated antiangiogenic response was suggested [56]. However, up to date no direct connection between FGFRs and galectins has been reported.

Our data define extracellular galectin-1 and -3 as novel FGFR1 binding proteins that directly interact with the sugar chains of the receptor. Although both galectins compete for the sugar residues on FGFR1, they elicit different effects on the receptor. Galectin-1 mimics FGF ligand, as it efficiently activates FGFR1 and initiates downstream signaling cascades resulting in cell proliferation and avoidance of apoptosis (Fig. 4h). In contrast, galectin-3 induces extensive cross-linking of FGFR1 on the cell surface, inhibiting constitutive receptor internalization (Fig. 4h). This differential effect of galectin-1 and galectin-3 can be attributed to the distinct receptor clustering properties of these proteins. Galectin-1 is relatively small dimeric protein, galectin-3 is considerably larger and assembles into pentamers [22]. Thus, it is highly probable that in galectin-1-induced clusters FGFR1 molecules are in close proximity, which permits autophosphorylation of intracellular kinase domains and initiates signaling (Fig. 4h). Similar activity of galectin-1 was suggested for other RTK member, VEGFR2 in endothelial cells [33]. FGFR1 molecules are more separated from each other when cross-linked by large, pentameric galectin-3, which results in only very weak activation of intracellular signaling cascades (Fig. 4h). Additionally, the differential effect of galectin-1 and galectin-3 on FGFR1 activity may result from their distinct binding kinetics, which may modulate the duration and dynamics of FGFR1-galectin complexes in the plasma membrane.

Numerous tumors secrete galectins that facilitate proliferation and migration of cancer cells [57, 58]. Our data suggest, that extracellular galectins may directly activate FGFR1 to fuel cancer cell division and survival. Noteworthy, galectin-1, galectin-3 and canonical FGFR1 ligands, FGF1 and FGF2 represent rare proteins that reach extracellular space via unconventional secretory mechanisms, further pointing on the functional interplay between galectins and FGFR signaling system [25, 59–62]. Summarizing, galectin-FGFRs interplay should be taken into consideration in the design of targeted anticancer therapies against cancers with dysregulated FGFRs.

## Additional file

Additional file 1: **Table S1.** Mass spectrometry experiments. Results of MS-based peptide identification of streptavidin-agarose pull down with U2OS-R1 and U2OS-SBP-R1 cells. **Figure S1.** Internalization of cell surface proteins upon FGF1 treatment. **Figure S2.** Interaction of endogenous galectin-1 and galectin-3 with FGFR1. **Figure S3.** Direct interaction of galectin-1 and -3 with FGFRs. **Figure S4.** Galectin-3-induced clustering of FGFR1 on the cell surface. **Figure S5.** Functionality tests of fluorescently labeled proteins. **Figure S6.** Functional interplay between galectin-1/− 3 and FGFR1. (DOCX 1060 kb)

## Abbreviations

BN-PAGE: Blue native polyacrylamide gel electrophoresis
CBB: Coomassie brilliant blue
CHO: Chinese hamster ovary cells
EDIL-3: EGF-like repeat and discoidin I-like domain-containing protein 3
EGFR: Epidermal growth factor receptor
ERK1/2: Extracellular signal-regulated kinases ½
FGFRs: Fibroblast growth factor receptors
FGFs: Fibroblast growth factors
GST: Glutathione S transferase
IR: Insulin receptor
MS: Mass spectrometry
PLCγ: Phospholipase C gamma
RSK1: Ribosomal S6-kinase
SBP: Streptavidin binding peptide
VAPA: Vesicle-associated membrane protein-associated protein A
VEGFR: Vascular endothelial growth factor receptor
