# Supplementary material for: Differential regulation of fibroblast growth factor receptor 1 trafficking and function by extracellular galectins
Source: Cell Commun Signal. 2019 Jun 17;17:65. doi: 10.1186/s12964-019-0371-1 (PMC6572767; doi:10.1186/s12964-019-0371-1)
Supplement: Supplementary file 1 — Table S1. Mass spectrometry experiments. Results of MS-based peptide identification of streptavidin-agarose pull down with U2OS-R1 and U2OS-SBP-R1 cells. Figure S1. Internalization of cell surface proteins upon FGF1 treatment. Figure S2. Interaction of endogenous galectin-1 and galectin-3 with FGFR1. Figure S3. Direct interaction of galectin-1 and -3 with FGFRs. Figure S4. Galectin-3-induced clustering of FGFR1 on the cell surface. Figure S5. Functionality tests of fluorescently labeled proteins. Figure S6. Functional interplay between galectin-1/− 3 and FGFR1. (DOCX 1060 kb) [file 12964_2019_371_MOESM1_ESM.docx]

**Differential regulation of FGFR1 trafficking and function by extracellular galectins**

by

Marika Kucińska, Natalia Porębska, Agata Lampart, Marta Latko, Agata Knapik, Małgorzata Zakrzewska, Jacek Otlewski and Łukasz Opaliński

**Additional file 1**

**Table of contents:**

1. Supplementary Methods

2. Supplementary Table

3. Supplementary Figure Legends

4. Supplementary Figures

**1. Supplementary Methods**

***Mass spectrometry experiments***

Beads with proteins were subjected to standard procedure of trypsin digestion, during which proteins were reduced with 5 mM TCEP for 1 h at 60°C, blocked with 10 mM MMTS for 10 min at RT and digested overnight with trypsin (0.1 mg/ml). Peptide mixtures were applied in equal volumes of 20 ul to RP-18 pre-column (Waters, Milford, MA) using water containing 0.1% FA as a mobile phase and then transferred to a nano-HPLC RP-18 column (internal diameter 75 µM, Waters, Milford MA) using ACN gradient (0 – 35% ACN in 160 min) in the presence of 0.1% FA at a flow rate of 250 nl/min. The column outlet was coupled directly to the ion source of the Q Exactive mass spectrometer (Thermo Electron Corp) measuring profile LC-MS spectra, without peptide fragmentation. A blank run ensuring absence of cross-contamination from previous samples preceded each analysis. Samples were measured in duplicate, once in data-dependent MS/MS mode and once as a profile LC-MS spectrum to acquire complete quantitative information.

The acquired MS/MS data from triplicates were preprocessed with Mascot Distiller software (v. 2.6, MatrixScience, London, UK) and a search was performed with the Mascot Search Engine (MatrixScience, London, UK, Mascot Server 2.5) against the human proteins deposited in Swissprot 2017_12 database (20,319 sequences) and Swissprot 2017_08 database (20,293 sequences).

To reduce mass errors, the peptide and fragment mass tolerance settings were established separately for individual LC-MS/MS runs after a measured mass recalibration, as described previously [[1](#_ENREF_1)]. The rest of search parameters were as follows: enzyme, Trypsin; missed cleavages, 1; fixed modifications, Methylthio (C); variable modifications, Oxidation (M); instrument, HCD. A statistical assessment of the confidence of peptide assignments was based on the target/decoy database search strategy [[2](#_ENREF_2)]. This procedure provided q-value estimates for each peptide spectrum match in the data set. All queries with q-values > 0.01 were removed from further analysis, as well as proteins with less than two peptides and proteins identified by a subset of peptides from another protein. Proteins that exactly matched the same set of peptides were combined into a single group (family). The mass calibration and data filtering described above were carried out with MScan software, developed in-house (<http://proteom.ibb.waw.pl/mscan/>).

The lists of identified peptides were merged into one common list. This list was overlaid onto 2-D heatmaps generated from LC-MS profile datasets by tagging the peptide-related isotopic envelopes with corresponding peptide sequence tags on the basis of the measured/theoretical mass difference, the deviation from the predicted elution time and the match between theoretical and observed isotopic envelopes. A more detailed description of the quantitative extraction procedure implemented by our in-house software is available in [[3](#_ENREF_3)]. The abundance of each peptide was determined as the height of a 2-D fit to the monoisotopic peak of the tagged isotopic envelope. Quantitative values were next exported into text files, along with peptide/protein identifications, for statistical analysis with Diffprot software [[1](#_ENREF_1)]. Diffprot was run with the following parameters: number of random peptide sets = 10 6 ; clustering of peptide sets – only when 90% identical; normalization by LOWESS.

***Immunoprecipitation***

NIH3T3 cells were washed with PBS and lysed in IP buffer (50 mM Tris pH 7.4, 150 mM NaCl, 1 mM EDTA, 0.2% Nonidet P-40, 1 mM PMSF, protease inhibitors cocktail) for 15 min on ice followed by brief sonication. Lysates were subsequently clarified by centrifugation (14000 rpm, 4⁰C, 10 min). Supernatants were incubated with 2-3 µg of appropriate antibody for 2h at 4⁰C and subsequently for 1h at RT. Immunocomplexes were captured on Protein-A Sepharose resin (1h, 4⁰C). Beads were extensively washed with PBS and bound proteins were eluted with SDS-PAGE sample buffer. Proteins were separated with SDS-PAGE and analyzed with western blotting.

**2. Supplementary Table**

**Introduction to Table S1**

Proteins identified in U2OS-SBP-R1 pull down and absent in U2OS-R1 pull down, and proteins enriched in U2OS-SBP-R1 experiments over U2OS-R1 pull down are listed in Table S1

**Table S1**. Results of MS-based peptide identification of streptavidin-agarose pull down with U2OS-R1 and U2OS-SBP-R1 cells.

| Position | Protein ID | Protein name | Score ((peptide matches) for each experiment repeat) or enrichment U2OS-SBP-R1 over U2OS-R1 | Reference  for confirmed interactions |
| --- | --- | --- | --- | --- |
|  | P11362 | FGF receptor 1 (FGFR1) | 3378.0 (30)  4164.0 (28)  5566.0 (42)  5067.0 (32)  4058.0 (28) | - |
| 1 | P51812 | Ribosomal protein S6 kinase alpha-3  (RSK-3) | 160.0 (4)  282.0 (7)  209.0 (5)  137.0 (3)  218.0 (5) | [[4](#_ENREF_4)] |
| 2 | Q16543 | Hsp90 co-chaperone Cdc37 | 150.0 (3)  190.0 (6)  104.0 (2)  84.0 (1)  270.0 (6) | [[5](#_ENREF_5)] |
| 3 | A6NHL2 | Tubulin alpha chain-like 3 | 190.0 (3)  266.0 (3)  245.0 (3)  141.0 (2)  263.0 (2) | - |
| 4 | P05230 | Fibroblast growth factor1  (FGF1) | 124.0 (2)  173.0 (2)  116.0 (2)  62.0 (1)  133.0 (2) | [[6](#_ENREF_6)] |
| 5 | P07237 | Protein disulfide-isomerase (P4HB) | 136.0 (3)  186.0 (4)  265.0 (4)  97.0 (2) | - |
| 6 | P30101 | Protein disulfide-isomerase A3 (PDIA3) | 113.0 (3)  78.0 (2)  82.0 (2)  102.0 (2) | - |
| 7 | Q9Y230 | RuvB-like 2 (RUVBL2) | 87.0 (2)  41.0 (1)  124.0 (3)  87.0 (2) | [[5](#_ENREF_5)] |
| 8 | Q86VP6 | Cullin-associated NEDD8-dissociated protein 1 (CAND1) | 83.0 (2)  45.0 (1)  140.0 (2)  121.0 (3) | - |
| 9 | P09038 | Fibroblast growth factor 2 (FGF2) | 96.0 (1)  120.0 (2)  202.0 (2)  56.0 (1) | [[7](#_ENREF_7)] |
| 10 | P06702 | Protein S100-A9 | 800.0 (6)  272.0 (5) | - |
| 11 | Q15418 | Ribosomal protein S6 kinase alpha-1 (RSK-1) | 107.0 (3)  158.0 (4) | [[8](#_ENREF_8)] |
| 12 | Q58FF6 | Putative heat shock protein HSP 90-beta 4 | 99.0 (2)  147.0 (3) | - |
| 13 | P63104 | 14-3-3 protein zeta/delta | 51.0 (1)  60.0 (2)  110.0 (3) | - |
| 14 | O00148/ Q13838 | Spliceosome RNA helicase DDX39A/B | 43.0 (1)  105.0 (3)  65.0 (2) | - |
| 15 | Q96P70 | Importin-9 | 58.0 (1)  74.0 (2)  115.0 (3) | - |
| 16 | Q13835 | Plakophilin-1 | 107.0 (2)  95.0 (2) | - |
| 17 | P33993 | DNA replication licensing factor MCM7 | 59.0 (1)  36.01 (1)  98.0 (3) | - |
| 18 | P41252 | Isoleucine--tRNA ligase | 81.0 (2)  59.0 (1)  85.0 (2) | - |
| 19 | P13929 | Beta-enolase | 265.0 (2)  56.0 (1)  124.0 (2) | - |
| 20 | O95470 | Sphingosine-1-phosphate lyase 1 | 49.0 (1)  85.0 (2) | - |
| 21 | O15260 | Surfeit locus protein 4 | 50.0 (1)  89.0 (2) | - |
| 22 | O60361 | Putative nucleoside diphosphate kinase | 85.0 (2)  58.0 (1) | - |
| 23 | Q8NBQ5 | Estradiol 17-beta-dehydrogenase 11 | 40.0 (1)  79.0 (2) | - |
| 24 | Q00325 | Phosphate carrier protein, mitochondrial | 49.0 (1)  63.0 (2) | - |
| 25 | Q9BWM7 | Sideroflexin-3 | 53.0 (1)  68.0 (2)  53.0 (1) | - |
| 26 | P61619/ Q9H9S3 | Protein transport protein Sec61 subunit alpha isoform 1/2 | 84.0 (2)  59.0 (1)  58.0 (1)  86.0 (2) | - |
| 27 | O95864 | Fatty acid desaturase 2 | 49.0 (1)  56.0 (1)  69.0 (2) | - |
| 28 | P49257 | Protein ERGIC-53 | 82.0 (2)  36.0 (1)  60.0 (1) | - |
| 29 | Q03252 | Lamin-B2 | 54.0 (1)  71.0 (2)  41.0 (1) | - |
| 30 | Q14108 | Lysosome membrane protein 2 | 91.0 (1)  59.0 (1)  73.0 (1) | - |
| 31 | P28066 | Proteasome subunit alpha type-5 | 50.0 (1)  37.0 (1)  43.0 (1) | - |
| 32 | P36404 | ADP-ribosylation factor-like protein 2 | 48.0 (1)  47.0 (1)  48.0 (1) | - |
| 33 | P36406 | ADP-ribosylation factor 6 | 43.0 (1)  60.0 (1)  43.0 (1) | [[9](#_ENREF_9)] |
| 34 | P39748 | Flap endonuclease 1 | 45.0 (1)  53.0 (1)  41.0 (1) | - |
| 35 | P47897 | Glutamine--tRNA ligase | 44.0 (1)  69.0 (2) | - |
| 36 | Q15758 | Neutral amino acid transporter B(0) | 45.0 (1)  37.0 (1)  45.0 (1)  50.0 (1) | [[9](#_ENREF_9)] |
| 37 | Q9BZG1 | Ras-related protein Rab-34 | 49.0 (1)  39.0 (1)  64.0 (1) | - |
| 38 | Q06210 | Glutamine--fructose-6-phosphate aminotransferase [isomerizing] 1 | 47.0 (1)  59.0 (1)  55.0 (1) | - |
| 39 | P53007 | Tricarboxylate transport protein, mitochondrial | 40.0 (1)  47.0 (1)  127.0 (3) | - |
| 40 | O43615 | Mitochondrial import inner membrane translocase subunit TIM44 | 67.0 (1)  76.0 (1)  55.0 (1) | - |
| 41 | O00264 | Membrane-associated progesterone receptor component 1 | 76.0 (1)  75.0 (1)  83.0 (1) | [[5](#_ENREF_5)] |
| 42 | O00232 | 26S proteasome non-ATPase regulatory subunit 12 | 56.0 (1)  46.0 (1)  41.0 (1) | - |
| 43 | O43854 | EGF-like repeat and discoidin I-like domain-containing protein 3 | 37.0 (1)  129.0 (2) | - |
| 44 | Q96BM9/ Q9NVJ2 | ADP-ribosylation factor-like protein 8A/B | 46.0 (1)  143.0 (3) | - |
| 45 | P51153 | Ras-related protein Rab-13 | 55.0 (1)  46.0 (1) | - |
| 46 | O14983 | Sarcoplasmic/endoplasmic reticulum calcium ATPase 1 | 90.0 (1)  49.0 (1) | - |
| 47 | P53618 | Coatomer subunit beta | 55.0 (1)  50.0 (1) | - |
| 48 | P67812 | Signal peptidase complex catalytic subunit SEC11A | 58.0 (1)  40.0 (1) | - |
| 49 | P69905 | Hemoglobin subunit alpha | 47.0 (1)  43.0 (1) | - |
| 50 | Q6UW68 | Transmembrane protein 205 | 50.0 (1)  43.0 (1) | - |
| 51 | Q8NC51 | Plasminogen activator inhibitor 1 RNA-binding protein | 51.0 (1)  49.0 (1) | - |
| 52 | Q8TC12 | Retinol dehydrogenase 11 | 73.0 (1)  39.0 (1) | - |
| 53 | Q8WWC4 | m-AAA protease-interacting protein 1, mitochondrial | 47.0 (1)  49.0 (1) | - |
| 54 | P22061 | Protein-L-isoaspartate(D-aspartate) O-methyltransferase | 48.0 (1)  48.0 (1) | - |
| 55 | Q99460 | 26S proteasome non-ATPase regulatory subunit 1 | 44.0 (1)  40.0 (1) | - |
| 56 | P16435 | NADPH--cytochrome P450 reductase | 46.0 (1)  37.0 (1) | [[9](#_ENREF_9)] |
| 57 | Q9H0U3 | Magnesium transporter protein 1 | 44.0 (1)  53.0 (1) | - |
| 58 | Q9BZZ5 | Apoptosis inhibitor 5 | 46.0 (1)  40.0 (1) | - |
| 59 | Q8IW41 | MAP kinase-activated protein kinase 5 | 41.0 (1)  39.0 (1) | - |
| 60 | P67809 | Nuclease-sensitive element-binding protein 1 | 46.0 (1)  46.0 (1) | - |
| 61 | O60884 | DnaJ homolog subfamily A member 2 | 50.0 (1)  45.0 (1) | - |
| 62 | Q08380 | Galectin-3-binding protein | 59.0 (1)  41.0 (1) | - |
| 63 | P46459 | Vesicle-fusing ATPase | 50.0 (1)  49.0 (1) | - |
| 64 | Q14739 | Lamin-B receptor | 47.0 (1)  47.0 (1) | - |
| 65 | Q16563 | Synaptophysin-like protein 1 | 50.0 (1)  45.0 (1) | - |
| 66 | P35052 | Glypican-1 | 54.0 (1)  52.0 (1) | - |
| 67 | P25205 | DNA replication licensing factor MCM3 | 49.0 (1)  43.0 (1) | - |
| 68 | Q8IXB1 | DnaJ homolog subfamily C member 10 | 45.0 (1)  41.0 (1) | - |
| 69 | Q9NV70 | Exocyst complex component 1 | 45.0 (1)  56.0 (1) | - |
| 70 | Q9P035 | Very-long-chain (3R)-3-hydroxyacyl-CoA dehydratase 3 | 46.0 (1)  37.0 (1) | - |
| 71 | Q9UHG3 | Prenylcysteine oxidase | 43.0 (1)  48.0 (1) | - |
| 72 | Q9Y266 | Nuclear migration protein nudC | 49.0 (1)  45.0 (1) | - |
| 73 | O96000 | NADH dehydrogenase [ubiquinone] 1 beta subcomplex subunit 10 | 46.0 (1)  51.0 (1) | - |
| 74 | P10301 | Ras-related protein R-Ras | 89.0 (1)  36.0 (1) | - |
| 75 | P08237 | ATP-dependent 6-phosphofructokinase, muscle type | 42.0 (1)  39.0 (1) | - |
| 76 | O75340 | Programmed cell death protein 6 | 42.0 (1)  44.0 (1) | - |
| 77 | O15269 | Serine palmitoyltransferase 1 | 49.0 (1)  38.0 (1) | - |
| 78 | Q9UBS4 | DnaJ homolog subfamily B member 11 | 50.0 (1)  47.0 (1) | - |
| 79 | Q13885 | Tubulin beta-2A chain | 2368.0 (14) | - |
| 80 | P47929 | Galectin-7 | 212.0 (3) | [[10](#_ENREF_10)] |
| 81 | Q71U36 | Tubulin alpha-1A chain | 1808.0 (15) | - |
| 82 | P78527 | DNA-dependent protein kinase catalytic subunit | 261.0 (6) | - |
| 83 | Q14240 | Eukaryotic initiation factor 4A-II | 274.0 (4) | - |
| 84 | P17066 | Heat shock 70 kDa protein 6 | 190.0 (3) | [[5](#_ENREF_5)] |
| 85 | P09104 | Gamma-enolase | 224.0 (3) | - |
| 86 | P84085 | ADP-ribosylation factor 5 | 200.0 (3) | - |
| 87 | Q15349 | Ribosomal protein S6 kinase alpha-2 (RSK-2) | 124.0 (3) | [[4](#_ENREF_4)] |
| 88 | P20337 | Ras-related protein Rab-3B | 150.0 (2) | - |
| 89 | P14314 | Glucosidase 2 subunit beta | 136.0 (3) | - |
| 90 | P31947 | 14-3-3 protein sigma | 114.0 (3) | - |
| 91 | Q9P0L0 | Vesicle-associated membrane protein-associated protein A (VAPA) | 99.0 (2) | - |
| 92 | P62266 | 40S ribosomal protein S23 | 92.0 (2) | - |
| 93 | P55072 | Transitional endoplasmic reticulum ATPase | 70.0 (2) | - |
| 94 | P61019 | Ras-related protein Rab-2A | 72.0 (2) | - |
| 95 | Q15293 | Reticulocalbin-1 | 76.0 (2) | - |
| 96 | Q8WUD1 | Ras-related protein Rab-2B | 74.0 (2) | [[5](#_ENREF_5)] |
| 97 | Q9UL25 | Ras-related protein Rab-21 | 96.0 (2) | - |
| 98 | Q05639 | Elongation factor 1-alpha 2 | 166.0 (4) | - |
| 99 | Q96IU4 | Protein ABHD14B | 86.0 (2) | - |
| 100 | Q13724 | Mannosyl-oligosaccharide glucosidase | 103.0 (2) | - |
| 101 | Q8WVV4 | Protein POF1B | 116.0 (2) | - |
| 102 | O60762 | Glial fibrillary acidic protein | 62.0 (2) | - |
| 103 | P11021 | 78 kDa glucose-regulated protein | 1.96 | - |
| 104 | Q13748 | Tubulin alpha 3C/ alpha 1A | 1.96 | - |
| 105 | P55084 | Trifunctional enzyme subunit beta, mitochondrial | 1.77 | - |
| 106 | P31689 | DnaJ homolog subfamily A member 1 | 1.75 | - |
| 107 | P27824 | Calnexin | 1.73 | [[5](#_ENREF_5)] |
| 108 | P07437 | Tubulin beta chain | 1.65 | - |
| 109 | P40939 | Trifunctional enzyme subunit alpha, mitochondrial | 1.48 | - |
| 110 | P17812 | CTP synthase 1 | 1.46 | - |
| 111 | O43175 | D-3-phosphoglycerate dehydrogenase | 1.39 | - |
| 112 | P49419 | Alpha-aminoadipic semialdehyde dehydrogenase | 1.38 | - |
| 113 | Q9H853 | Putative tubulin-like protein alpha-4B | 1.36 | - |
| 114 | P14625 | Endoplasmin | 1.33 | - |
| 115 | P04843 | Dolichyl-diphosphooligosaccharide--protein glycosyltransferase subunit 1 | 1.33 | - |
| 116 | P08238 | Heat shock protein HSP 90-beta | 1.32 | - |
| 117 | Q9BSJ8 | Extended synaptotagmin-1 | 1.3 | - |
| 118 | Q9BUF5 | Tubulin beta-6 chain | 1.28 | - |
| 119 | P50454 | Serpin H1 | 1.25 | - |
| 120 | P05141 | ADP/ATP translocase 2 | 1.25 | - |
| 121 | P07900 | Heat shock protein HSP 90-alpha | 1.24 | [[11](#_ENREF_11)] |
| 122 | P09382 | Galectin-1 | 1.24 | - |
| 123 | Q3ZCQ8 | Mitochondrial import inner membrane translocase subunit TIM50 | 1.21 | - |

**Supplementary References:**

1. Malinowska A, Kistowski M, Bakun M, Rubel T, Tkaczyk M, Mierzejewska J, Dadlez M (2012) Diffprot - software for non-parametric statistical analysis of differential proteomics data. *J Proteomics* **75**: 4062-73

2. Elias JE, Haas W, Faherty BK, Gygi SP (2005) Comparative evaluation of mass spectrometry platforms used in large-scale proteomics investigations. *Nat Methods* **2**: 667-75

3. Bakun M, Karczmarski J, Poznanski J, Rubel T, Rozga M, Malinowska A, Sands D, Hennig E, Oledzki J, Ostrowski J*, et al.* (2009) An integrated LC-ESI-MS platform for quantitation of serum peptide ladders. Application for colon carcinoma study. *Proteomics Clin Appl* **3**: 932-46

4. Nadratowska-Wesolowska B, Haugsten EM, Zakrzewska M, Jakimowicz P, Zhen Y, Pajdzik D, Wesche J, Wiedlocha A (2014) RSK2 regulates endocytosis of FGF receptor 1 by phosphorylation on serine 789. *Oncogene* **33**: 4823-36

5. Vecchione A, Cooper HJ, Trim KJ, Akbarzadeh S, Heath JK, Wheldon LM (2007) Protein partners in the life history of activated fibroblast growth factor receptors. *Proteomics* **7**: 4565-78

6. Levi E, Fridman R, Miao HQ, Ma YS, Yayon A, Vlodavsky I (1996) Matrix metalloproteinase 2 releases active soluble ectodomain of fibroblast growth factor receptor 1. *Proc Natl Acad Sci U S A* **93**: 7069-74

7. Pantoliano MW, Horlick RA, Springer BA, Van Dyk DE, Tobery T, Wetmore DR, Lear JD, Nahapetian AT, Bradley JD, Sisk WP (1994) Multivalent ligand-receptor binding interactions in the fibroblast growth factor system produce a cooperative growth factor and heparin mechanism for receptor dimerization. *Biochemistry* **33**: 10229-48

8. Hu Y, Fang X, Dunham SM, Prada C, Stachowiak EK, Stachowiak MK (2004) 90-kDa ribosomal S6 kinase is a direct target for the nuclear fibroblast growth factor receptor 1 (FGFR1): role in FGFR1 signaling. *J Biol Chem* **279**: 29325-35

9. Kostas M, Haugsten EM, Zhen Y, Sorensen V, Szybowska P, Fiorito E, Lorenz S, Jones N, de Souza GA, Wiedlocha A*, et al.* (2018) Protein Tyrosine Phosphatase Receptor Type G (PTPRG) Controls Fibroblast Growth Factor Receptor (FGFR) 1 Activity and Influences Sensitivity to FGFR Kinase Inhibitors. *Mol Cell Proteomics* **17**: 850-870

10. Huttlin EL, Ting L, Bruckner RJ, Gebreab F, Gygi MP, Szpyt J, Tam S, Zarraga G, Colby G, Baltier K*, et al.* (2015) The BioPlex Network: A Systematic Exploration of the Human Interactome. *Cell* **162**: 425-440

11. Taipale M, Krykbaeva I, Koeva M, Kayatekin C, Westover KD, Karras GI, Lindquist S (2012) Quantitative analysis of HSP90-client interactions reveals principles of substrate recognition. *Cell* **150**: 987-1001

**3. Supplementary Figure Legends**

**Introduction to Figure S1**

**A**. U2OS-SBP-R1 cells were surface-biotinylated with EZsulfo-SS-biotin and treated with FGF1 (100 ng/ml) and heparin (10 U/ml) for various time points. Biotinylated proteins were labeled with Streptavidin-AlexaFluor-555 and visualized with fluorescence microscopy. Scale bars represent 20 µm. **B**. FGF1 induces internalization of SBP-FGFR1. U2OS-SBP-R1 cells were incubated with Streptavidin-AlexaFluor-555 to mark SBP-FGFR1 and left untreated or stimulated with FGF1 (100 ng/ml) and heparin (10 U/ml). Nuclei were labeled and cells were analyzed with fluorescence microscopy. Scale bars represent 50 µm. **C**. Quantification of internalized SBP-FGFR1 or cell surface proteins from three experiments of A and B. Average values +/- SEM are shown. Student t test was used for statistical analysis (* p<0.05).

**Figure S1. Internalization of cell surface proteins upon FGF1 treatment**.

**Introduction to Figure S2**

Co-IP experiments of NIH3T3 cell lysates incubated with anti-FGFR1 (A) or anti-galectin-1 and anti-galectin-3 antibodies (B). Proteins co-purified with specific antibodies were analyzed with western blotting.

**Figure S2. Interaction of endogenous galectin-1 and galectin-3 with FGFR1**.

**Introduction to Figure S3**

**A.** SDS-PAGE analysis of recombinant, purified galectin-1 and galectin-3. Proteins were visualized with CBB. **B**. Western blotting analysis of purity of U2OS-SBP-R1 fractions subjected to pull down in Fig. 2D. **C**. Immunofluorescence analysis of galectin-1 localization in U2OS-SBP-R1 cells. Scale bar represents 50 µm. **D** and **E**. BLI analyses of FGFR2-FGFR4-galectin interaction. The extracellular part of FGFR2-FGFR4 (FGFR2ecd-Fc-FGFR4ecd-Fc) (10 µg/ml) was immobilized of Protein-A sensors and interaction with galectin-1 and galectin-3 (10-30 µg/ml) was determined.

**Figure S3. Direct interaction of galectin-1 and -3 with FGFRs.**

**Introduction to Figure S4**

U2OS-SBP-R1 cells were incubated with recombinant galectin-3 (10 µg/ml) for 10 min at 37⁰C. Cells were subsequently fixed, SBP-FGFR1 was labeled with Streptavidin DyLight550 and cell surface with CellMask Green. Nuclei were stained with NucLive Blue and cells were analyzed with fluorescence microscopy. Scale bar represents 20 µm.

**Figure S4. Galectin-3-induced clustering of FGFR1 on the cell surface**.

**Introduction to Figure S5**

BLI analyses of FGFR1ecd-Fc interaction with fluorescently labeled FGF2, galectin-1 and galectin-3. Non-labeled proteins were used as a controls. FGFR1ecd-Fc (10 µg/ml) was immobilized on Protein-A sensors and incubated with studied proteins (10-30 µg/ml).

**Figure S5. Functionality tests of fluorescently labeled proteins.**

**Introduction to Figure S6**

**A.** Extended classification of cells into various clusters from Fig. 4D. **B**. Anti-apoptotic activity of galectin proteins assessed in NIH3T3 cells with caspase-3/7 activity measurements. Cells were subjected to serum starvation for 24 h to induce apoptosis. Cells were treated with FGF1 (200 ng/ml), galectin-1 and galectin-3 (10µg/ml) and heparin (10 U/ml) in the presence or absence of FGFR inhibitor (100 nM PD173074) for 16 h. Next, caspase-3/7 activity was and normalized to cells untreated with FGF1 and treated with PD173074. Average values from 3 experiments +/-SD are shown. **C**. Galectin-1 induced cell proliferation depends on FGFR1 activation. Galectin-1 induced cell proliferation was assessed as in Fig. 4G, but in the presence of FGFR inhibitor PD173074 (100 nM). Results were normalized to the cell response triggered by FGF1. Average values from at least 3 experiments +/-SEM are shown.

**Figure S6. Functional interplay between galectin-1/-3 and FGFR1.**

**4. Supplementary Figures**

**
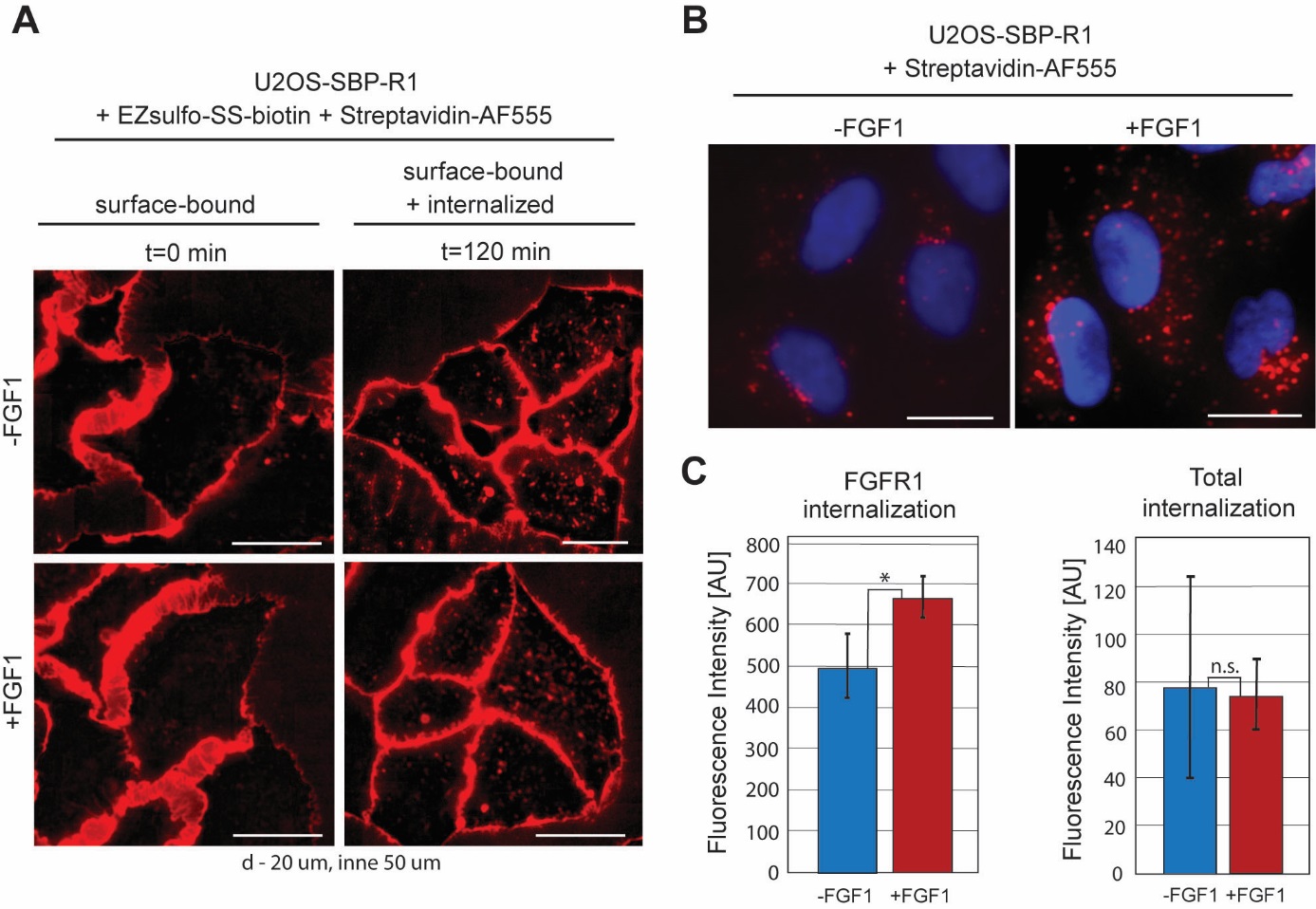
**

**Figure S1**

**
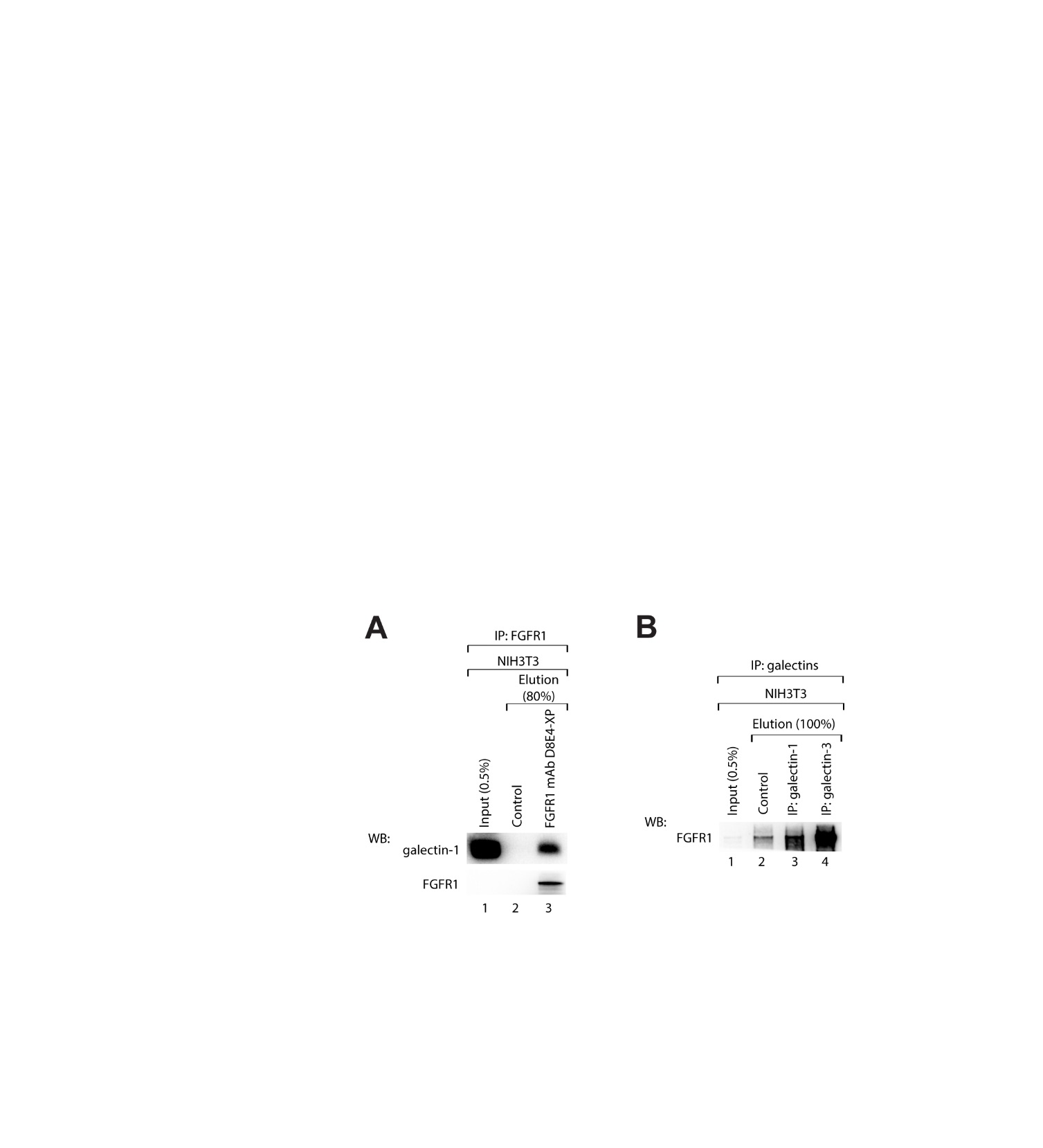
**

**Figure S2**

**
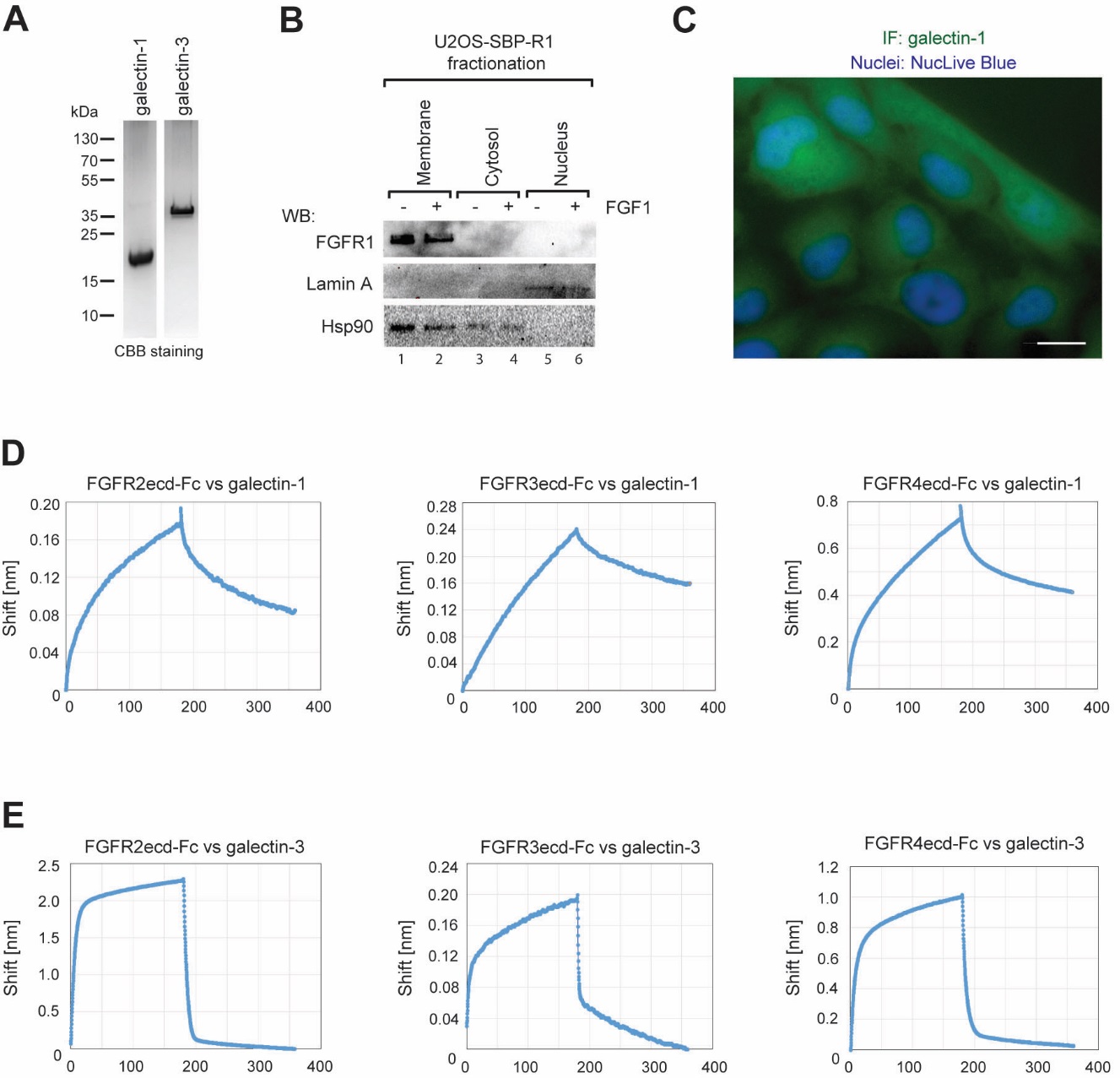
**

**Figure S3**

**
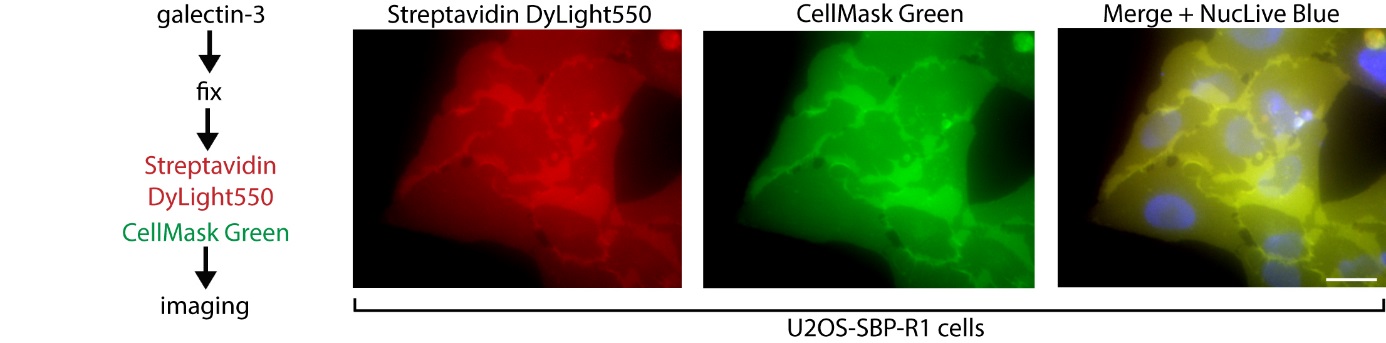
**

**Figure S4**

**
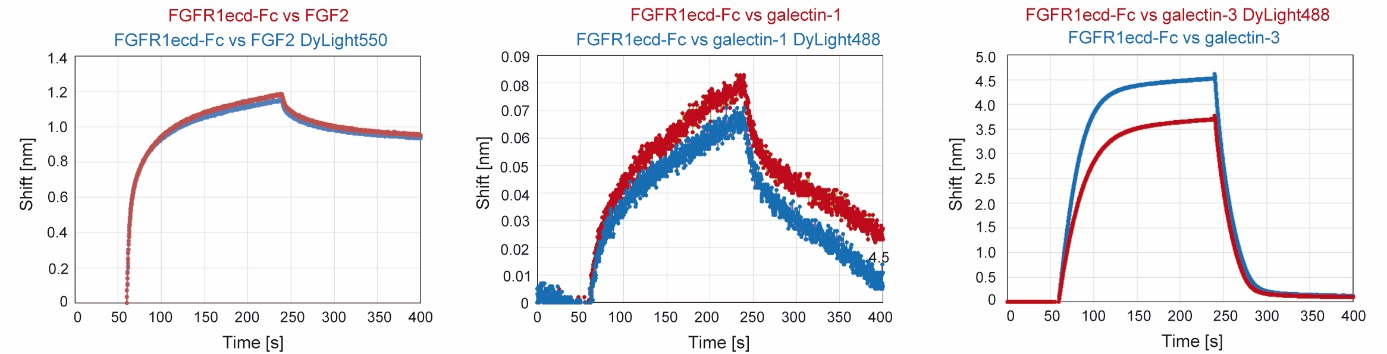
**

**Figure S5**

**
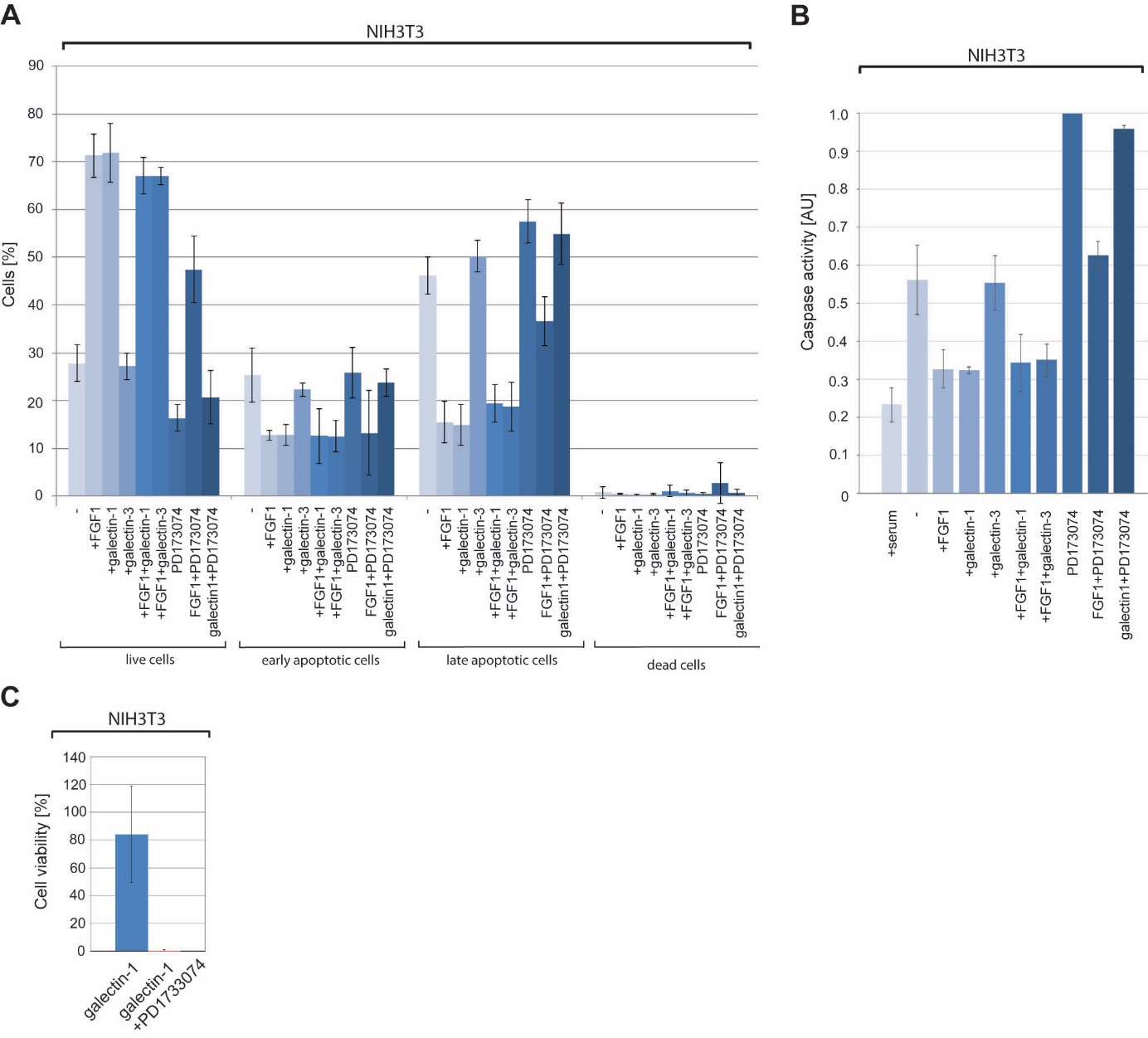
**

**Figure S6**
